# Analysis of flow rate and pressure in syringe-based wound irrigation using Bernoulli's equation

**DOI:** 10.1038/s41598-022-19402-2

**Published:** 2022-09-02

**Authors:** Hanna Lee, Ye-kyung Lee, Ji-Yun Park, Jeong-won Han

**Affiliations:** 1 Gangneung-Wonju National University, Wonju-si, Gangwon-do Republic of Korea; 2 College of Nursing Science, Kyung Hee University, Seoul, Republic of Korea

**Keywords:** Health care, Health services

## Abstract

The objective of this study was to examine the dependence of the pressure level in the wound area on the height of the syringe needle from the wound, the gauge of the needle, and the flow rate using the Bernoulli equation. This study was the control-volume analysis using the Bernoulli equation. At a given height of the syringe needle from the wound, the gauge of the syringe needle was fixed, and the pressure in the wound area, which depended on the flow rate of the irrigation solution discharged from the tip of the needle, was calculated according to the Bernoulli equation and the definition of the flow rate. At a constant flow rate of the irrigation solution, the velocity of the irrigation solution discharged through the syringe needle decreased (7.80 → 0.80) with an increase in the diameter of the needle (18G → 14G). At a constant inner diameter of the needle, the velocity of the irrigation solution increased with a reduction in the flow rate of the solution. As the velocity of the irrigation solution increased, the pressure in the wound area increased. As the height of the syringe needle from the wound area increased, the pressure in the wound area increased. In order to maintain the pressure of 8–15 psi when nurses perform syringe-based irrigation, it is necessary to set the flow rate of the cleaning solution from 3.5 cc/s to less than 4.8 cc/s for 19G. In addition, 20G maintains the flow rate of the solution at 2.6 cc/s or more and less than 3.5 cc/s, 22G maintains the flow rate of solution at 1.3 cc/s or more and less than 1.8 cc/s, and 25G maintains the flow rate of solution at 0.5 cc/s. This study provides nurses with a reference for the flow rate at which syringe-based irrigation can be performed while maintaining the appropriate pressure based on fluid dynamics, which can be used as the basis for wound nursing standards.

## Introduction

According to the 2020 health insurance statistics, the medical costs for domestic chronic diseases increased by 0.5% in 2020 compared with 2019^1^. Medical costs for chronic diseases are increasing, and a wound occurring in a chronic-disease patient may require a long recovery time or become chronic owing to the nature of the disease, leading to long-term use of medical services and higher medical costs^2,3^. Therefore, many countries are focusing on the role of nurses in the primary medical system for the management of chronic patients. In the United States, patient-centered medical homes and nurse-managed health centers are operated, and in the United Kingdom, nurses are responsible for managing patients' chronic and common diseases, vaccinations, and healthcare^4^. In South Korea, a home nursing system is operated, and nurses are in charge of various direct and indirect nursing services^5^. Wound management is a common responsibility of primary and home care nurses domestically and internationally, and many professional institutions and associations provide wound management education to nurses.

Wound management follows the following fundamental principles. First, the wound is evaluated, foreign substances are removed if applicable, and wound irrigation is performed as necessary^6^. Wound irrigation involves the use of an irrigation solution to remove foreign substances attached to the wound surface by applying a constant pressure to the surface. This reduces the bacterial load and infection rate and plays an important role in improving the wound environment and healing the wound by preventing and delaying the formation of a biofilm^7^. The Agency of Health Care Policy and Study (AHCPR) recommends an irrigation pressure of 10 pounds per square inch (psi) ~ 15 psi. The removal of bacteria can be less effective if the irrigation is performed at a pressure lower than 10 psi ~ 15 psi, whereas a pressure exceeding 15 psi may damage the healthy tissue of the patient and cause pain^8^.

Various medical instruments have been developed and clinically used to maintain adequate pressure for wound irrigation, but the products are expensive and difficult to disinfect, store, and reuse. Additionally, it is difficult to maintain a pressure of 8 psi ~ 15 psi even with these medical instruments^9^. Thus, medical professionals are still using syringe-based irrigation (syringe needle irrigation), which is inexpensive and convenient. In syringe-based irrigation, it is possible to maintain the pressure at 8 psi with a 19-gauge needle in a 35 cc syringe and at 13 psi with a 22-gauge needle in a 12 cc syringe^10^. However, a recent study indicated that syringe-based irrigation performed by healthcare professionals is more suitable for maintaining a pressure of 8 psi ~ 15 psi than irrigation with other instruments designed to maintain a constant pressure but has a larger pressure deviation every time the irrigation is performed on a patient^11^. To address this limitation, it is necessary to provide nurses with a reference for the flow rate at which syringe-based irrigation can be performed while maintaining the appropriate pressure.

In this regard, fluid dynamics have recently been applied in various fields, including medicine and mechanics, in addition to engineering. From the perspective of fluid dynamics, all substances are classified into fluids and solids. A fluid can be described as an object whose strain can be mathematically or quantitatively determined according to the shear stress under the assumption that the fluid is composed of a continuous body^12^. Fluid dynamics are applied in various fields because there are no limitations on the sizes or locations of the analysis subjects. In general, it is expensive and time-consuming to establish a perfect experimental environment, but fluid engineering has the advantage of allowing calculations without an experimental structure while achieving excellent accuracy, as it involves no experimental errors^13^.

In fluid dynamics, the movement of a fluid is called a fluid flow, which can be generally classified as a laminar flow or a turbulent flow depending on the flow characteristics. In a laminar flow, the viscous force is more dominant than the inertial force, and fluid particles with a low density and high viscosity move in an orderly manner while maintaining a uniform layer at a low velocity. In a turbulent flow, the inertial force is more dominant than the viscous force, and fluid particles with a relatively high density and low viscosity move at a high velocity while changing and mixing irregularly^14^. Although various criteria are available for distinguishing laminar and turbulent flows, the Reynolds number [$\mathrm{Re}$, Eq. (1)] is generally used. A small Reynolds number indicates laminar flow characteristics, whereas a large Reynolds number indicates turbulent flow characteristics^15^.

1 $${\text{Re}} = \frac{\rho VD}{\mu }$$

Here, $\rho$ represents the fluid density, $V$ represents the characteristic velocity of the fluid, $\mu$ represents the kinematic viscosity, and $D$ represents the characteristic length. The following two methods can be used for analyzing a fluid flow. The first method is to analyze the pressure and velocity at which the fluid properties can be defined for all locations and times of a flow field. In this method, which is called differential analysis, the continuity equation and the Navier–Stokes equation governing the fluid motion are solved to analyze the fluid flow^16^. The second method, which is called control-volume analysis, is to analyze the flow by defining a finite area within the flow field and applying the laws of mass conservation and momentum conservation to the finite area^15^. A control volume is an arbitrary area in the space where the fluid can move. If an appropriate control volume is selected, the pressure and stress applied to the control volume can be analyzed, and the velocity and flow rate of the fluid passing through the control volume can be determined^17^. The control-volume analysis method is widely used not only in engineering but also in the medical field because it allows the flow characteristics to be analyzed relatively easily. Bernoulli's equation—one of the popular equations in the field of fluid dynamics—can be used for control-volume analysis.

Bernoulli's equation, which describes the relationships among the pressure, kinetic energy, and potential energy for a fluid flow, is expressed as follows:

2 $$p + \frac{{\rho V^{2} }}{2} + \rho gz = constant,$$

where $p$ represents the pressure, $z$ represents the height, and $g$ represents the gravitational acceleration, which was 9.81 m/s^2^ in this study.

In contrast to the conventional method of selecting the pressure according to the syringe size and needle gauge, the following research problem is examined using the Bernoulli equation of fluid dynamics, and supporting data for syringe-based irrigation are presented.

Research question: When a nurse performs syringe-based irrigation, what is the appropriate flow rate for the given gauge of the syringe needle and distance from the wound to maintain a pressure of 8 psi ~ 15 psi in the wound?

## Results

### Pressure in wound area depending on flow rate and height of irrigation solution

**Figure 1:**
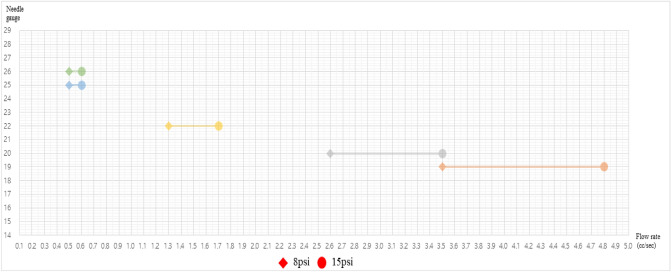
Flow rate range by syringe gauge.

**Figure 2:**
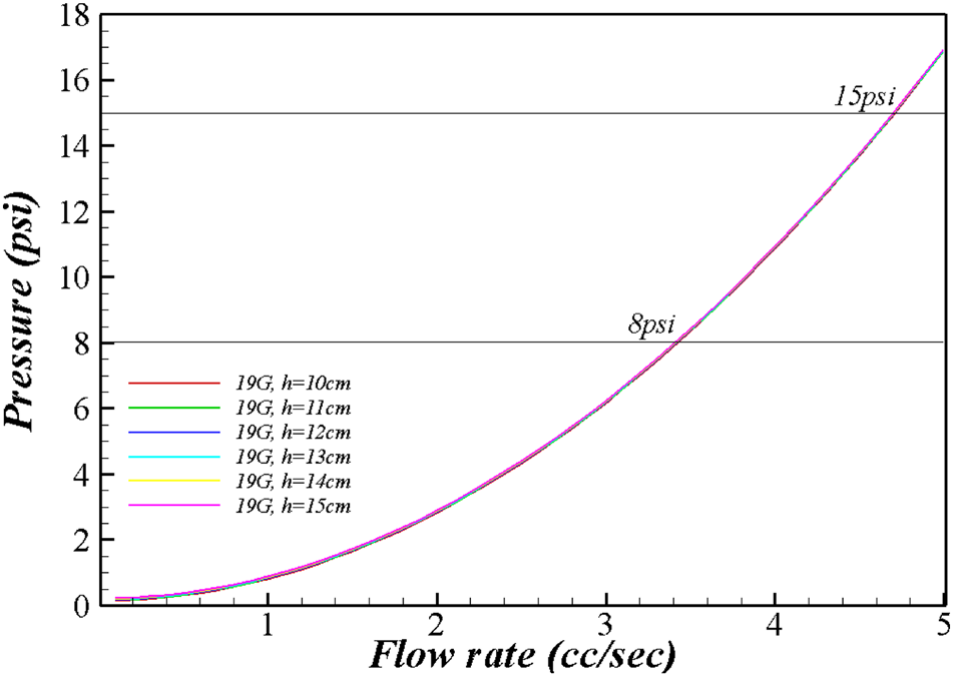
Range of flow rate and pressure for 19 gauge.

**Figure 3:**
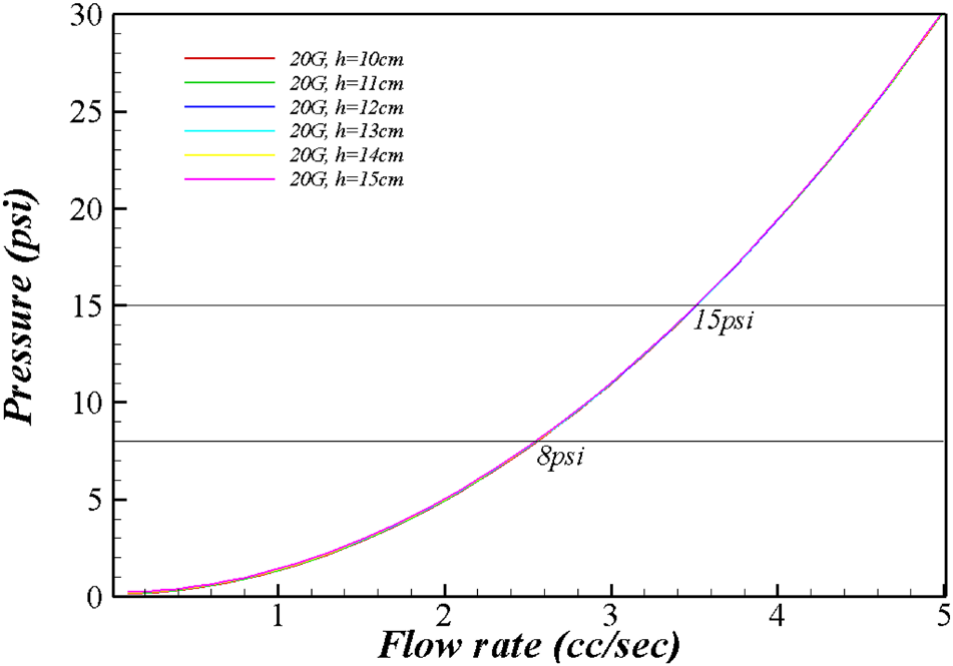
Range of flow rate and pressure for 20 gauge.

Tables 1, 2, 3 and Fig. 1, 2, 3, 4 present the pressure in the wound area with respect to the flow rate and height of the irrigation solution, and Supplements 1 and 2 show the detailed results. Examining the pressure in the wound area with respect to the flow rate and the height of the irrigation solution for each syringe needle gauge revealed that the maximum pressure in the wound area was 0.80 psi for 14G at the maximum irrigation-solution flow rate of 5.0 cc/s and the maximum height of 15 cm. At the same maximum irrigation-solution flow rate of 5.0 cc/s and height of 15 cm, the maximum pressure in the wound area was 2.23 psi for 16G, 3.84 psi for 17G, and 7.80 psi for 18G.

**Table 1:** Flow rate analysis according to the height from the wound of 19 gauge.

Flow rate (cc/s)	h (cm)					
	10	11	12	13	14	15
0.1	0.150	0.164	0.178	0.192	0.207	0.221
0.2	0.170	0.184	0.198	0.213	0.227	0.241
0.3	0.203	0.217	0.232	0.246	0.260	0.275
0.4	0.250	0.264	0.279	0.293	0.307	0.321
0.5	0.310	0.325	0.339	0.353	0.367	0.382
0.6	0.384	0.398	0.413	0.427	0.441	0.455
0.7	0.471	0.485	0.500	0.514	0.528	0.542
0.8	0.571	0.586	0.600	0.614	0.629	0.643
0.9	0.685	0.700	0.714	0.728	0.742	0.757
1.0	0.813	0.827	0.841	0.855	0.870	0.884
1.1	0.953	0.967	0.982	0.996	1.010	1.025
1.2	1.107	1.121	1.136	1.150	1.164	1.179
1.3	1.275	1.289	1.303	1.317	1.332	1.346
1.4	1.455	1.470	1.484	1.498	1.513	1.527
1.5	1.650	1.664	1.678	1.692	1.707	1.721
1.6	1.857	1.872	1.886	1.900	1.914	1.929
1.7	2.078	2.092	2.107	2.121	2.135	2.150
1.8	2.313	2.327	2.341	2.355	2.370	2.384
1.9	2.560	2.575	2.589	2.603	2.618	2.632
2.0	2.822	2.836	2.850	2.864	2.879	2.893
2.1	3.096	3.110	3.125	3.139	3.153	3.168
2.2	3.384	3.398	3.413	3.427	3.441	3.455
2.3	3.685	3.700	3.714	3.728	3.743	3.757
2.4	4.000	4.014	4.029	4.043	4.057	4.072
2.5	4.328	4.343	4.357	4.371	4.385	4.400
2.6	4.670	4.684	4.698	4.713	4.727	4.741
2.7	5.025	5.039	5.053	5.068	5.082	5.096
2.8	5.393	5.407	5.422	5.436	5.450	5.464
2.9	5.775	5.789	5.803	5.818	5.832	5.846
3.0	6.170	6.184	6.198	6.213	6.227	6.241
3.1	6.578	6.593	6.607	6.621	6.635	6.650
3.2	7.000	7.014	7.029	7.043	7.057	7.072
3.3	7.435	7.450	7.464	7.478	7.493	7.507
3.4	7.884	7.898	7.913	7.927	7.941	7.956
3.5	**8.346**	**8.360**	**8.375**	**8.389**	**8.403**	**8.418**
3.6	**8.822**	**8.836**	**8.850**	**8.865**	**8.879**	**8.893**
3.7	**9.310**	**9.325**	**9.339**	**9.353**	**9.368**	**9.382**
3.8	**9.813**	**9.827**	**9.841**	**9.856**	**9.870**	**9.884**
3.9	**10.328**	**10.343**	**10.357**	**10.371**	**10.386**	**10.400**
4.0	**10.857**	**10.872**	**10.886**	**10.900**	**10.915**	**10.929**
4.1	**11.400**	**11.414**	**11.428**	**11.443**	**11.457**	**11.471**
4.2	**11.956**	**11.970**	**11.984**	**11.998**	**12.013**	**12.027**
4.3	**12.525**	**12.539**	**12.553**	**12.568**	**12.582**	**12.596**
4.4	**13.107**	**13.122**	**13.136**	**13.150**	**13.165**	**13.179**
4.5	**13.703**	**13.718**	**13.732**	**13.746**	**13.761**	**13.775**
4.6	**14.313**	**14.327**	**14.341**	**14.356**	**14.370**	**14.384**
4.7	**14.936**	**14.950**	**14.964**	**14.978**	**14.993**	15.007
4.8	15.572	15.586	15.600	15.615	15.629	15.643
4.9	16.221	16.236	16.250	16.264	16.278	16.293
5.0	16.884	16.899	16.913	16.927	16.941	16.956

Significant values are in bold.

**Table 2:** Flow rate analysis according to the height from the wound of 20 gauge.

Flow rate (cc/s)	h (cm)					
	10	11	12	13	14	15
0.1	0.155	0.169	0.183	0.198	0.212	0.226
0.2	0.191	0.205	0.220	0.234	0.248	0.262
0.3	0.251	0.265	0.280	0.294	0.308	0.322
0.4	0.335	0.349	0.364	0.378	0.392	0.406
0.5	0.443	0.457	0.472	0.486	0.500	0.514
0.6	0.575	0.589	0.604	0.618	0.632	0.647
0.7	0.731	0.745	0.760	0.774	0.788	0.803
0.8	0.911	0.926	0.940	0.954	0.968	0.983
0.9	1.115	1.130	1.144	1.158	1.173	1.187
1.0	1.344	1.358	1.372	1.386	1.401	1.415
1.1	1.596	1.610	1.624	1.638	1.653	1.667
1.2	1.872	1.886	1.900	1.915	1.929	1.943
1.3	2.172	2.186	2.200	2.215	2.229	2.243
1.4	2.496	2.510	2.525	2.539	2.553	2.568
1.5	2.844	2.859	2.873	2.887	2.901	2.916
1.6	3.216	3.231	3.245	3.259	3.274	3.288
1.7	3.613	3.627	3.641	3.656	3.670	3.684
1.8	4.033	4.047	4.061	4.076	4.090	4.104
1.9	4.477	4.491	4.506	4.520	4.534	4.549
2.0	4.945	4.960	4.974	4.988	5.002	5.017
2.1	5.438	5.452	5.466	5.480	5.495	5.509
2.2	5.954	5.968	5.982	5.997	6.011	6.025
2.3	6.494	6.508	6.523	6.537	6.551	6.566
2.4	7.058	7.073	7.087	7.101	7.116	7.130
2.5	7.647	7.661	7.675	7.690	7.704	7.718
2.6	**8.259**	**8.273**	**8.288**	**8.302**	**8.316**	**8.330**
2.7	**8.895**	**8.910**	**8.924**	**8.938**	**8.952**	**8.967**
2.8	**9.556**	**9.570**	**9.584**	**9.598**	**9.613**	**9.627**
2.9	**10.240**	**10.254**	**10.269**	**10.283**	**10.297**	**10.311**
3.0	**10.948**	**10.963**	**10.977**	**10.991**	**11.005**	**11.020**
3.1	**11.681**	**11.695**	**11.709**	**11.724**	**11.738**	**11.752**
3.2	**12.437**	**12.451**	**12.466**	**12.480**	**12.494**	**12.509**
3.3	**13.217**	**13.232**	**13.246**	**13.260**	**13.275**	**13.289**
3.4	**14.022**	**14.036**	**14.050**	**14.065**	**14.079**	**14.093**
3.5	**14.850**	**14.865**	**14.879**	**14.893**	**14.907**	**14.922**
3.6	15.703	15.717	15.731	15.746	15.760	15.774
3.7	16.579	16.593	16.608	16.622	16.636	16.651
3.8	17.480	17.494	17.508	17.522	17.537	17.551
3.9	18.404	18.418	18.433	18.447	18.461	18.476
4.0	19.353	19.367	19.381	19.395	19.410	19.424
4.1	20.325	20.339	20.354	20.368	20.382	20.396
4.2	21.322	21.336	21.350	21.364	21.379	21.393
4.3	22.342	22.356	22.371	22.385	22.399	22.413
4.4	23.387	23.401	23.415	23.429	23.444	23.458
4.5	24.455	24.469	24.484	24.498	24.512	24.527
4.6	25.548	25.562	25.576	25.591	25.605	25.619
4.7	26.664	26.679	26.693	26.707	26.721	26.736
4.8	27.805	27.819	27.833	27.848	27.862	27.876
4.9	28.969	28.984	28.998	29.012	29.027	29.041
5.0	30.158	30.172	30.187	30.201	30.215	30.229

Significant values are in bold.

**Table 3:** Flow rate analysis according to the height from the wound of 22–27 gauge.

Flow rate (cc/s)	h (cm)					
	10	11	12	13	14	15
**22 gauge**						
1.0	5.247	5.261	5.275	5.290	5.304	5.318
1.1	6.318	6.333	6.347	6.361	6.376	6.390
1.2	7.492	7.507	7.521	7.535	7.550	7.564
1.3	**8.768**	**8.783**	**8.797**	**8.811**	**8.825**	**8.840**
1.4	**10.146**	**10.161**	**10.175**	**10.189**	**10.203**	**10.218**
1.5	**11.626**	**11.641**	**11.655**	**11.669**	**11.684**	**11.698**
1.6	**13.209**	**13.223**	**13.237**	**13.251**	**13.266**	**13.280**
1.7	**14.893**	**14.907**	**14.921**	**14.936**	**14.950**	**14.964**
1.8	16.679	16.693	16.708	16.722	16.736	16.751
1.9	18.568	18.582	18.596	18.610	18.625	18.639
2.0	20.558	20.572	20.587	20.601	20.615	20.630
**25 gauge**						
0.1	0.550	0.565	0.579	0.593	0.608	0.622
0.2	1.773	1.787	1.802	1.816	1.830	1.845
0.3	3.811	3.825	3.840	3.854	3.868	3.882
0.4	6.664	6.678	6.693	6.707	6.721	6.735
0.5	**10.332**	**10.346**	**10.361**	**10.375**	**10.389**	**10.404**
0.6	**14.815**	**14.830**	**14.844**	**14.858**	**14.872**	**14.887**
0.7	20.114	20.128	20.142	20.157	20.171	20.185
0.8	26.227	26.242	26.256	26.270	26.284	26.299
0.9	33.156	33.170	33.184	33.199	33.213	33.227
1.0	40.900	40.914	40.928	40.943	40.957	40.971
**26 gauge**						
0.1	0.550	0.565	0.579	0.593	0.608	0.622
0.2	1.773	1.787	1.802	1.816	1.830	1.845
0.3	3.811	3.825	3.840	3.854	3.868	3.882
0.4	6.664	6.678	6.693	6.707	6.721	6.735
0.5	**10.332**	**10.346**	**10.361**	**10.375**	**10.389**	**10.404**
0.6	**14.815**	**14.830**	**14.844**	**14.858**	**14.872**	**14.887**
0.7	20.114	20.128	20.142	20.157	20.171	20.185
0.8	26.227	26.242	26.256	26.270	26.284	26.299
0.9	33.156	33.170	33.184	33.199	33.213	33.227
1.0	40.900	40.914	40.928	40.943	40.957	40.971
**27 gauge**						
0.1	1.173	1.187	1.202	1.216	1.230	1.244
0.2	4.263	4.278	4.292	4.306	4.320	4.335
0.3	**9.414**	**9.428**	**9.442**	**9.457**	**9.471**	**9.485**
0.4	16.625	16.639	16.653	16.667	16.682	16.696
0.5	25.895	25.910	25.924	25.938	25.953	25.967
0.6	37.227	37.241	37.255	37.269	37.284	37.298
0.7	50.618	50.632	50.646	50.661	50.675	50.689
0.8	66.069	66.084	66.098	66.112	66.127	66.141
0.9	83.581	83.595	83.610	83.624	83.638	83.653
1.0	103.153	103.167	103.182	103.196	103.210	103.225

Significant values are in bold.

**Figure 4:**
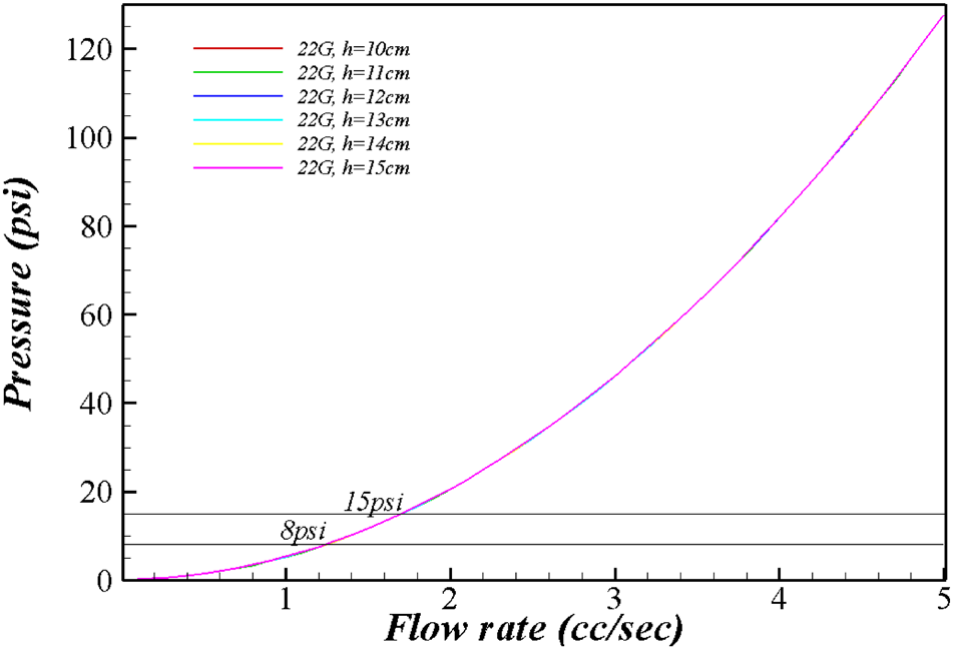
Range of flow rate and pressure for 22 gauge.

The pressure in the wound area for 19G was calculated to be ≥ 8.0 psi at irrigation-solution flow rates of ≥ 3.5 cc/s, and it increased to ≥ 15.0 psi at irrigation-solution flow rates of ≥ 4.8 cc/s. The pressure in the wound area for 20G was ≥ 8.0 psi at irrigation-solution flow rates of ≥ 2.6 cc/s, and it increased to ≥ 15.0 psi at irrigation-solution flow rates of ≥ 3.5 cc/s. The pressure in the wound area for 22G was ≥ 8.0 psi at irrigation-solution flow rates of ≥ 1.3 cc/s, and it increased to ≥ 15.0 psi at irrigation-solution flow rates of ≥ 1.8 cc/s.

The pressure in the wound area was ≥ 10 psi for 25G even at an irrigation-solution flow rate of 0.5 cc/s and a height of 10 cm (the lowest rate and height considered in this study). The pressure in the wound area was 14.8 psi ~ 14.9 psi (depending on the height) at the irrigation-solution flow rate of 0.6 cc/s, and it increased to ≥ 20 psi at irrigation-solution flow rates of ≥ 0.7 cc/s. The pressure in the wound area was ≥ 25 and ≥ 94 psi for 27G and 29G, respectively, even at the minimum irrigation-solution flow rate of 0.5 cc/s and the minimum height of 10 cm.

In this study, the Reynolds number based on the diameter of the needle and the flow rate of the irrigation solution at the needle is 419-47,200. The case with a 29 gauge needle and flow rate of 5 cc/s has the largest Reynolds number, and a 14 gauge needle with a flow rate of 0.5 cc/s has the smallest Reynolds number.

### Characteristics of irrigation-solution flow rate, velocity of irrigation solution discharged at needle, height, and pressure in wound area

At a constant flow rate of the irrigation solution, the velocity of the irrigation solution discharged through the syringe needle decreased as the needle diameter increased. According to the law of mass preservation, the velocity of the irrigation solution discharged through the needle was inversely proportional to the square of the needle diameter.

At a constant inner diameter of the syringe needle, the velocity of the irrigation solution discharged through the needle increased with the flow rate of the irrigation solution. According to the law of mass conservation, the velocity of the irrigation solution discharged through the needle had a linear relationship with the flow rate of the irrigation solution; i.e., when the flow rate doubled, the velocity of the irrigation solution discharged through the needle also doubled.

As the velocity of the irrigation solution increased, the pressure in the wound area increased. According to the Bernoulli equation, the pressure in the wound area was proportional to the square of the velocity of the irrigation solution discharged through the needle. Therefore, the pressure in the wound area with the changing flow rate of the irrigation solution was expressed as a secondary function by setting the x variable as the flow rate and the y variable as the pressure in the wound area.

As the height of the syringe needle from the wound area increased, the pressure in the wound area increased. According to the Bernoulli equation, the height and the pressure in the wound area had a linear relationship. Considering (3), the relationship between the flow rate of the irrigation solution and the pressure in the wound area was expressed as a secondary function. As the height increased, the secondary function moved toward the y-axis, and the distance moved was proportional to the height. A recent study indicated that syringe-based irrigation performed by healthcare professionals is more suitable for maintaining a pressure of 8 psi ~ 15 psi than other instruments designed to maintain a constant pressure but has a larger pressure deviation every time the irrigation is performed on a patient^11^. The results of the present study provide supporting evidence for the method to compensate for this downside of syringe-based irrigation.

## Discussion

The objective of this study was to apply fluid dynamics and provide nurses with a standard for the flow rate at which syringe-based irrigation can be performed while maintaining the appropriate pressure. The results are discussed in this section.

First, it was found that at a constant flow rate of the irrigation solution, the velocity of the irrigation solution discharged through the needle decreases as the diameter of the syringe needle increases, and at a constant inner diameter of the needle, the velocity of the irrigation solution discharged through the needle increases as the flow rate of the irrigation solution increases. These results differ from those of previous studies and guidelines; it was previously reported that pressures of 8 and 13 psi can be achieved using a 19G needle in a 35 cc syringe and a 22-gauge needle in a 12 cc syringe, respectively, during syringe-based wound irrigation^10^. These previous studies and wound nursing guidelines suggest a combination of the syringe size and needle diameter to maintain the specified pressure, but the results of the present study indicate that the pressure is determined by the diameter of the needle rather than by the syringe size. In a previous study^18^, a pressure of 8 psi was generated with a 19G catheter and a 35 mL syringe; however, in the present study, the pressure was < 8.0 psi even with a 19G needle when the flow rate of the irrigation solution was ≤ 3.5 cc/s, whereas the pressure was ≥ 15.0 psi at an irrigation-solution flow rate of 4.8 cc/s. These results indicate that for the same needle diameter, the pressure depends on the flow rate. Additionally, even at the maximum flow rate suggested in this study, it was difficult to maintain the pressure of 8 psi ~ 15 psi required for wound irrigation using a needle gauge between 14 and 18G. These results indicate that a nurse should determine the flow rate for syringe-based wound irrigation according to the appropriate syringe needle gauge.

Second, in this study, the pressure in the wound area increased with the velocity of the irrigation solution and the height of the needle from the wound area. In the existing guidelines for syringe-based wound irrigation, the devices for irrigation are specified to be placed 10–15 cm (4–6 inches) from the wound^18,19^. However, as demonstrated in this study, the pressure depends on the height of the syringe needle, and it is recommended that nurses consider the syringe needle gauge and the height from the wound for maintaining the appropriate pressure required for patients during syringe-based wound irrigation.

Third, the results of this study confirmed that the range of the flow rate yielding a pressure of 8 psi ~ 15 psi varied with respect to the gauge of the syringe needle. As the needle gauge decreased, this range narrowed. The range of the flow rate for a 25G catheter was narrow (0.1–0.2 cc/s) because the pressure in the wound was ≥ 10 psi at the irrigation-solution flow rate of 0.5 cc/s and the height of 10 cm, while the pressure in the wound was ≥ 20 psi at irrigation-solution flow rates of ≥ 0.7 cc/s. Nurses should consider the dependence of the flow-rate range on the needle diameter during syringe-based wound irrigation.

In conclusion, this study, it was demonstrated that the needle diameter, flow rate of the irrigation solution, and height of the needle from the wound surface should be considered by nurses to maintain the appropriate pressure of 8 psi ~ 15 psi when performing syringe-based wound irrigation. Therefore, in order for a nurse to maintain a pressure of 8–15 psi at a distance of 10–15 cm from the wound when a nurse performs syringe-based irrigation, the flow rate of the irrigation solution of 19G should be 3.5 cc/s or more and less than 4.8 cc/s, 2.6 cc/s or more and less than 3.5 cc/s for 20G, 1.3 cc/s or more and less than 1.8 cc/s for 22G, and 0.5 cc/s or more and less than 0.6 cc/s for 25G. However, a needle gauge of less than 18G is challenging to apply a pressure of 8psi or more to the wound. In addition, because syringe needles over 27G exceed pressures over 15 psi, nurses should be careful in selecting a syringe needle of ≥ 27G and ≤ 18G during wound irrigation. The results of this study can facilitate the development of work standards for improving the quality of wound care, and the criteria for syringe-based wound irrigation identified in this study can be used for training nurses and nursing students. Additionally, from the viewpoint of nursing practice, the results of this study can be applied to the management of syringe-based wound irrigation for developing efficient and effective methods for resources while enhancing the quality of nursing. However, the limitations of this study are as follows. The limitation of the analysis method using Bernoulli's equation is that it cannot consider friction loss. However, wound irrigation is described as an external flow, and with no friction due to solid contact, such as internal flow. The friction that may occur in this study may include friction due to surface tension. However, since the irrigation solution is continuously sprayed, the surface tension effect is negligible except for the initial spraying. Therefore, it is considered that the flow rate analysis of syringe-based wound irrigation using the Bernoulli equation used in this study has sufficient clinical application value. The following suggestion is made for further research. First, while the control-volume method was used in this study for syringe-based wound irrigation, it is recommended to apply computational fluid dynamics for examining the relationship between the pressure and the flow rate in wound irrigation to enhance the accuracy and validity of the results. In addition, in future research, we propose an experimental study using the results of computational fluid dynamics by manufacturing a machine that can directly measure the flow rate and pressure using a syringe needle and irrigation solution. Moreover, it is necessary to study the cost-effectiveness and effectiveness of performing the task of wound care based on this study. Lastly, we will propose an ISO standard for syringe-based wound irrigation manual and related device after conducting an expansion study based on this study.

## Methods

### Control-volume analysis

**Figure 5:**
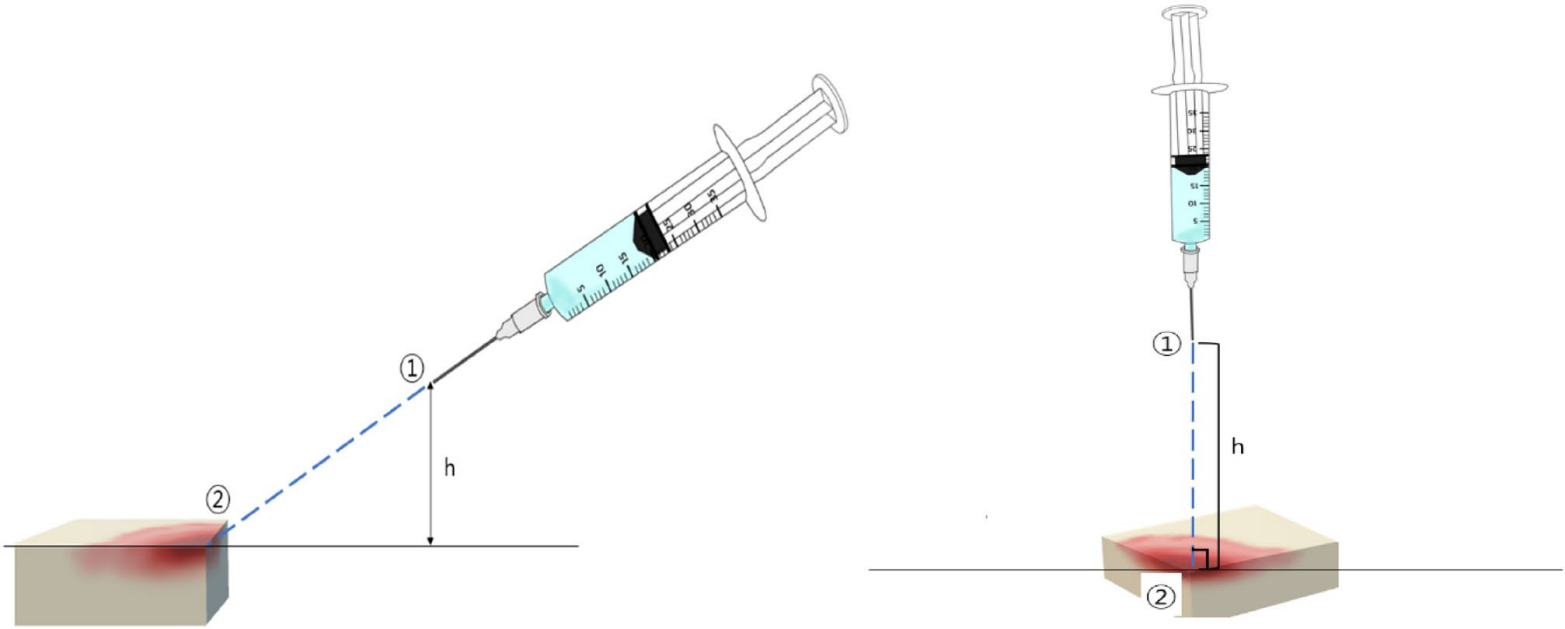
Structure of analyze system.

In this study, the control-volume analysis method was employed using the Bernoulli equation to determine the pressure in the wound area depending on the height of the syringe needle from the wound, the gauge of the syringe needle, and the flow rate during the syringe-based irrigation. The outline of the system to be analyzed in this study is as follows (Fig. 5). Since this study is not an experimental study on humans or animals, obtaining consent from the research subjects is not an applicable but theoretical study, and it was conducted upon exemption from review by the Gangneung-Wonju National University Institutional Review Board (GWNUIRB-R2022-40). It was conducted in compliance with the IRB's research performance guideline.

### Configuration of control volume

In this study, the control volume spans from the tip of the syringe needle (①), through which the irrigation solution is discharged, to the surface of the wound (②). The components of the control volume are as follows.

1. Syringe needle

The inner diameter of the syringe needle suggested by ISO differs according to normal-walled, thin-walled, and extra-thin-walled. In this study, normal-walled was used as the standard. In this study, the inner diameter of the syringe needle (normal-walled) specified by the International Organization for Standardization (ISO) was used in the calculations. The gauges of the syringe needles were set as 14G, 16G, 17G, 18G, 19G, 20G, 22G, 25G, 27G, and 29G. The internal diameters of the syringe needles based on the ISO standard were found to be 29G (0.133 mm), 27G (0.184 mm), 25G (0.232 mm), 22G (0.390 mm), 20G (0.560 mm), 19G (0.648 mm), 18G (0.790 mm), 17G (0.950 mm), 16G (1.100 mm), and 14G (1.500 mm).

2. Irrigation solution

Among the various irrigation solutions available for wound irrigation, the commonly used 0.9% saline solution at room temperature (20 °C) was selected. The density of the 0.9% saline solution was 1.0046 g/mL.

3. Height of syringe needle from wound (h; height)

In this study, the height of the syringe needle from the wound area was set as 10–15 cm according to the guidelines of the British Columbia Provincial Nursing Skin & Wound Committee^20^.

### Assumptions

In this study, the Bernoulli equation was applied under the assumptions of a steady state, incompressibility, a frictionless flow, and a single streamline. It was also assumed that the prerequisite of no external transfer of energy or heat was satisfied. Subscript 1 in Eq. (3) refers to the top of the syringe needle, corresponding to point ① in Fig. 5. Subscript 2 refers to the surface of the wound, corresponding to point ② in Fig. 5. ${p}_{1}$ represents the pressure at the location where the irrigation solution is sprayed into the air, which is equivalent to the atmospheric pressure under the jet condition. Because the relative pressure is used for the atmospheric pressure, ${p}_{1}$ = 0. ${V}_{2}$ represents the velocity at the surface of the wound, which can be set as ${V}_{2}$ = 0 under the no-slip condition. ${z}_{2}$ represents the height in the direction opposite to gravity, and ${z}_{2}$ = 0 at the reference position of the wound surface. ${z}_{1}$ represents the tip of the syringe needle, which increases from 10 to 15 cm in intervals of 1 cm. ${V}_{1}$ represents the velocity at which the irrigation solution passes through the tip of the syringe needle, which can be calculated using the mass conservation of the syringe system. Because the flow rate ($\mathrm{Q}$) of the solution pressed out by the syringe piston and the flow rate out of the syringe needle are conserved, ${V}_{1}$ can be calculated using the cross-sectional area (${A}_{1}$) of the syringe needle (Eq. (4)). In this study, ${V}_{1}$ was calculated while increasing the flow rate ($\mathrm{Q}$) of the irrigation solution from 0.5 to 5 cc/sec in intervals of 0.1 cc/sec and is then substituted into the Bernoulli equation.

3 $$p_{1} + \frac{{\rho V_{1}^{2} }}{2} + \rho gz_{1} = p_{2} + \frac{{\rho V_{2}^{2} }}{2} + \rho gz_{2}$$

4 $${\text{Q}} = V_{1} A_{1} = \frac{\pi }{4}D^{2}$$

### Analysis

The units for the irrigation solution and the internal diameter of the syringe needle were converted to the International System of Units (SI), and the pressure in the wound area, which depended on the flow rate of the irrigation solution discharged from the tip of the needle, was calculated using the Bernoulli equation (Eq. (3)) and the definition of the flow rate (Eq. (4)). In the equation for the flow rate ($\mathrm{Q}$) of the irrigation solution, $D$ represents the diameter of each syringe needle gauge. Although pressure loss occurs inside the needle, the pressure loss inside the needle is excluded from our study because the pressure becomes atmospheric pressure when the irrigation solution exits the needle.

## Supplementary Information

Supplementary Information 1.

Supplementary Information 2.

## Data Availability

The datasets used and/or analysed during the current study available from the corresponding author on reasonable request.
