# Supplementary material for: Analysis of flow rate and pressure in syringe-based wound irrigation using Bernoulli's equation
Source: Sci Rep. 2022 Sep 2;12:14957. doi: 10.1038/s41598-022-19402-2 (PMC9440234; doi:10.1038/s41598-022-19402-2)
Supplement: Supplementary file 1 — Supplementary Information 1. [file 41598_2022_19402_MOESM1_ESM.docx]

**Supplement 1. Flow rate analysis according to the height from the wound by syringe needle gauge**

<14G>

| Flow rate  (cc/sec) | h (cm) | | | | | |
| --- | --- | --- | --- | --- | --- | --- |
|  | 10 | 11 | 12 | 13 | 14 | 15 |
| 0.1 | 0.143 | 0.157 | 0.172 | 0.186 | 0.200 | 0.215 |
| 0.2 | 0.144 | 0.158 | 0.172 | 0.187 | 0.201 | 0.215 |
| 0.3 | 0.145 | 0.159 | 0.174 | 0.188 | 0.202 | 0.216 |
| 0.4 | 0.147 | 0.161 | 0.175 | 0.190 | 0.204 | 0.218 |
| 0.5 | 0.149 | 0.163 | 0.177 | 0.192 | 0.206 | 0.220 |
| 0.6 | 0.151 | 0.166 | 0.180 | 0.194 | 0.208 | 0.223 |
| 0.7 | 0.154 | 0.169 | 0.183 | 0.197 | 0.211 | 0.226 |
| 0.8 | 0.158 | 0.172 | 0.186 | 0.201 | 0.215 | 0.229 |
| 0.9 | 0.162 | 0.176 | 0.190 | 0.205 | 0.219 | 0.233 |
| 1.0 | 0.166 | 0.181 | 0.195 | 0.209 | 0.223 | 0.238 |
| 1.1 | 0.171 | 0.185 | 0.200 | 0.214 | 0.228 | 0.243 |
| 1.2 | 0.176 | 0.191 | 0.205 | 0.219 | 0.234 | 0.248 |
| 1.3 | 0.182 | 0.197 | 0.211 | 0.225 | 0.239 | 0.254 |
| 1.4 | 0.189 | 0.203 | 0.217 | 0.231 | 0.246 | 0.260 |
| 1.5 | 0.195 | 0.210 | 0.224 | 0.238 | 0.253 | 0.267 |
| 1.6 | 0.203 | 0.217 | 0.231 | 0.245 | 0.260 | 0.274 |
| 1.7 | 0.210 | 0.225 | 0.239 | 0.253 | 0.267 | 0.282 |
| 1.8 | 0.218 | 0.233 | 0.247 | 0.261 | 0.276 | 0.290 |
| 1.9 | 0.227 | 0.241 | 0.256 | 0.270 | 0.284 | 0.299 |
| 2.0 | 0.236 | 0.250 | 0.265 | 0.279 | 0.293 | 0.308 |
| 2.1 | 0.246 | 0.260 | 0.274 | 0.289 | 0.303 | 0.317 |
| 2.2 | 0.256 | 0.270 | 0.284 | 0.299 | 0.313 | 0.327 |
| 2.3 | 0.266 | 0.281 | 0.295 | 0.309 | 0.323 | 0.338 |
| 2.4 | 0.277 | 0.292 | 0.306 | 0.320 | 0.334 | 0.349 |
| 2.5 | 0.289 | 0.303 | 0.317 | 0.332 | 0.346 | 0.360 |
| 2.6 | 0.301 | 0.315 | 0.329 | 0.343 | 0.358 | 0.372 |
| 2.7 | 0.313 | 0.327 | 0.342 | 0.356 | 0.370 | 0.384 |
| 2.8 | 0.326 | 0.340 | 0.354 | 0.369 | 0.383 | 0.397 |
| 2.9 | 0.339 | 0.353 | 0.368 | 0.382 | 0.396 | 0.410 |
| 3.0 | 0.353 | 0.367 | 0.381 | 0.396 | 0.410 | 0.424 |
| 3.1 | 0.367 | 0.381 | 0.396 | 0.410 | 0.424 | 0.438 |
| 3.2 | 0.382 | 0.396 | 0.410 | 0.425 | 0.439 | 0.453 |
| 3.3 | 0.397 | 0.411 | 0.425 | 0.440 | 0.454 | 0.468 |
| 3.4 | 0.413 | 0.427 | 0.441 | 0.455 | 0.470 | 0.484 |
| 3.5 | 0.429 | 0.443 | 0.457 | 0.471 | 0.486 | 0.500 |
| 3.6 | 0.445 | 0.459 | 0.474 | 0.488 | 0.502 | 0.517 |
| 3.7 | 0.462 | 0.476 | 0.491 | 0.505 | 0.519 | 0.534 |
| 3.8 | 0.480 | 0.494 | 0.508 | 0.523 | 0.537 | 0.551 |
| 3.9 | 0.498 | 0.512 | 0.526 | 0.541 | 0.555 | 0.569 |
| 4.0 | 0.516 | 0.530 | 0.545 | 0.559 | 0.573 | 0.588 |
| 4.1 | 0.535 | 0.549 | 0.564 | 0.578 | 0.592 | 0.606 |
| 4.2 | 0.554 | 0.569 | 0.583 | 0.597 | 0.611 | 0.626 |
| 4.3 | 0.574 | 0.588 | 0.603 | 0.617 | 0.631 | 0.646 |
| 4.4 | 0.594 | 0.609 | 0.623 | 0.637 | 0.652 | 0.666 |
| 4.5 | 0.615 | 0.629 | 0.644 | 0.658 | 0.672 | 0.687 |
| 4.6 | 0.636 | 0.651 | 0.665 | 0.679 | 0.694 | 0.708 |
| 4.7 | 0.658 | 0.672 | 0.687 | 0.701 | 0.715 | 0.730 |
| 4.8 | 0.680 | 0.695 | 0.709 | 0.723 | 0.737 | 0.752 |
| 4.9 | 0.703 | 0.717 | 0.731 | 0.746 | 0.760 | 0.774 |
| 5.0 | 0.726 | 0.740 | 0.755 | 0.769 | 0.783 | 0.797 |

<16G>

| Flow rate  (cc/sec) | h (cm) | | | | | |
| --- | --- | --- | --- | --- | --- | --- |
|  | 10 | 11 | 12 | 13 | 14 | 15 |
| 0.1 | 0.144 | 0.158 | 0.172 | 0.187 | 0.201 | 0.215 |
| 0.2 | 0.146 | 0.160 | 0.175 | 0.189 | 0.203 | 0.218 |
| 0.3 | 0.150 | 0.164 | 0.179 | 0.193 | 0.207 | 0.222 |
| 0.4 | 0.156 | 0.170 | 0.184 | 0.199 | 0.213 | 0.227 |
| 0.5 | 0.163 | 0.177 | 0.192 | 0.206 | 0.220 | 0.235 |
| 0.6 | 0.172 | 0.186 | 0.201 | 0.215 | 0.229 | 0.243 |
| 0.7 | 0.182 | 0.197 | 0.211 | 0.225 | 0.240 | 0.254 |
| 0.8 | 0.195 | 0.209 | 0.223 | 0.237 | 0.252 | 0.266 |
| 0.9 | 0.208 | 0.223 | 0.237 | 0.251 | 0.265 | 0.280 |
| 1.0 | 0.224 | 0.238 | 0.252 | 0.266 | 0.281 | 0.295 |
| 1.1 | 0.240 | 0.255 | 0.269 | 0.283 | 0.298 | 0.312 |
| 1.2 | 0.259 | 0.273 | 0.288 | 0.302 | 0.316 | 0.330 |
| 1.3 | 0.279 | 0.293 | 0.308 | 0.322 | 0.336 | 0.351 |
| 1.4 | 0.301 | 0.315 | 0.330 | 0.344 | 0.358 | 0.372 |
| 1.5 | 0.324 | 0.339 | 0.353 | 0.367 | 0.382 | 0.396 |
| 1.6 | 0.349 | 0.364 | 0.378 | 0.392 | 0.407 | 0.421 |
| 1.7 | 0.376 | 0.390 | 0.405 | 0.419 | 0.433 | 0.447 |
| 1.8 | 0.404 | 0.418 | 0.433 | 0.447 | 0.461 | 0.476 |
| 1.9 | 0.434 | 0.448 | 0.463 | 0.477 | 0.491 | 0.505 |
| 2.0 | 0.465 | 0.480 | 0.494 | 0.508 | 0.523 | 0.537 |
| 2.1 | 0.499 | 0.513 | 0.527 | 0.541 | 0.556 | 0.570 |
| 2.2 | 0.533 | 0.548 | 0.562 | 0.576 | 0.590 | 0.605 |
| 2.3 | 0.570 | 0.584 | 0.598 | 0.612 | 0.627 | 0.641 |
| 2.4 | 0.607 | 0.622 | 0.636 | 0.650 | 0.665 | 0.679 |
| 2.5 | 0.647 | 0.661 | 0.676 | 0.690 | 0.704 | 0.718 |
| 2.6 | 0.688 | 0.702 | 0.717 | 0.731 | 0.745 | 0.760 |
| 2.7 | 0.731 | 0.745 | 0.759 | 0.774 | 0.788 | 0.802 |
| 2.8 | 0.775 | 0.789 | 0.804 | 0.818 | 0.832 | 0.847 |
| 2.9 | 0.821 | 0.835 | 0.850 | 0.864 | 0.878 | 0.893 |
| 3.0 | 0.869 | 0.883 | 0.897 | 0.912 | 0.926 | 0.940 |
| 3.1 | 0.918 | 0.932 | 0.946 | 0.961 | 0.975 | 0.989 |
| 3.2 | 0.969 | 0.983 | 0.997 | 1.012 | 1.026 | 1.040 |
| 3.3 | 1.021 | 1.035 | 1.050 | 1.064 | 1.078 | 1.093 |
| 3.4 | 1.075 | 1.089 | 1.104 | 1.118 | 1.132 | 1.147 |
| 3.5 | 1.131 | 1.145 | 1.159 | 1.174 | 1.188 | 1.202 |
| 3.6 | 1.188 | 1.202 | 1.217 | 1.231 | 1.245 | 1.260 |
| 3.7 | 1.247 | 1.261 | 1.276 | 1.290 | 1.304 | 1.318 |
| 3.8 | 1.307 | 1.322 | 1.336 | 1.350 | 1.365 | 1.379 |
| 3.9 | 1.370 | 1.384 | 1.398 | 1.412 | 1.427 | 1.441 |
| 4.0 | 1.433 | 1.448 | 1.462 | 1.476 | 1.490 | 1.505 |
| 4.1 | 1.499 | 1.513 | 1.527 | 1.541 | 1.556 | 1.570 |
| 4.2 | 1.565 | 1.580 | 1.594 | 1.608 | 1.623 | 1.637 |
| 4.3 | 1.634 | 1.648 | 1.663 | 1.677 | 1.691 | 1.705 |
| 4.4 | 1.704 | 1.718 | 1.733 | 1.747 | 1.761 | 1.776 |
| 4.5 | 1.776 | 1.790 | 1.805 | 1.819 | 1.833 | 1.847 |
| 4.6 | 1.849 | 1.864 | 1.878 | 1.892 | 1.907 | 1.921 |
| 4.7 | 1.924 | 1.939 | 1.953 | 1.967 | 1.982 | 1.996 |
| 4.8 | 2.001 | 2.015 | 2.030 | 2.044 | 2.058 | 2.072 |
| 4.9 | 2.079 | 2.093 | 2.108 | 2.122 | 2.136 | 2.151 |
| 5.0 | 2.159 | 2.173 | 2.188 | 2.202 | 2.216 | 2.230 |

<17G>

| Flow rate  (cc/sec) | h (cm) | | | | | |
| --- | --- | --- | --- | --- | --- | --- |
|  | 10 | 11 | 12 | 13 | 14 | 15 |
| 0.1 | 0.144 | 0.159 | 0.173 | 0.187 | 0.202 | 0.216 |
| 0.2 | 0.149 | 0.163 | 0.177 | 0.192 | 0.206 | 0.220 |
| 0.3 | 0.156 | 0.170 | 0.185 | 0.199 | 0.213 | 0.227 |
| 0.4 | 0.166 | 0.180 | 0.195 | 0.209 | 0.223 | 0.238 |
| 0.5 | 0.179 | 0.193 | 0.208 | 0.222 | 0.236 | 0.251 |
| 0.6 | 0.195 | 0.209 | 0.224 | 0.238 | 0.252 | 0.267 |
| 0.7 | 0.214 | 0.228 | 0.243 | 0.257 | 0.271 | 0.285 |
| 0.8 | 0.236 | 0.250 | 0.264 | 0.279 | 0.293 | 0.307 |
| 0.9 | 0.260 | 0.275 | 0.289 | 0.303 | 0.317 | 0.332 |
| 1.0 | 0.288 | 0.302 | 0.316 | 0.331 | 0.345 | 0.359 |
| 1.1 | 0.318 | 0.333 | 0.347 | 0.361 | 0.375 | 0.390 |
| 1.2 | 0.352 | 0.366 | 0.380 | 0.395 | 0.409 | 0.423 |
| 1.3 | 0.388 | 0.402 | 0.416 | 0.431 | 0.445 | 0.459 |
| 1.4 | 0.427 | 0.441 | 0.456 | 0.470 | 0.484 | 0.498 |
| 1.5 | 0.469 | 0.483 | 0.498 | 0.512 | 0.526 | 0.541 |
| 1.6 | 0.514 | 0.528 | 0.543 | 0.557 | 0.571 | 0.585 |
| 1.7 | 0.562 | 0.576 | 0.590 | 0.605 | 0.619 | 0.633 |
| 1.8 | 0.613 | 0.627 | 0.641 | 0.655 | 0.670 | 0.684 |
| 1.9 | 0.666 | 0.681 | 0.695 | 0.709 | 0.723 | 0.738 |
| 2.0 | 0.723 | 0.737 | 0.751 | 0.766 | 0.780 | 0.794 |
| 2.1 | 0.782 | 0.796 | 0.811 | 0.825 | 0.839 | 0.854 |
| 2.2 | 0.845 | 0.859 | 0.873 | 0.887 | 0.902 | 0.916 |
| 2.3 | 0.910 | 0.924 | 0.938 | 0.953 | 0.967 | 0.981 |
| 2.4 | 0.978 | 0.992 | 1.006 | 1.021 | 1.035 | 1.049 |
| 2.5 | 1.049 | 1.063 | 1.077 | 1.092 | 1.106 | 1.120 |
| 2.6 | 1.123 | 1.137 | 1.151 | 1.166 | 1.180 | 1.194 |
| 2.7 | 1.200 | 1.214 | 1.228 | 1.243 | 1.257 | 1.271 |
| 2.8 | 1.279 | 1.294 | 1.308 | 1.322 | 1.337 | 1.351 |
| 2.9 | 1.362 | 1.376 | 1.391 | 1.405 | 1.419 | 1.433 |
| 3.0 | 1.448 | 1.462 | 1.476 | 1.490 | 1.505 | 1.519 |
| 3.1 | 1.536 | 1.550 | 1.565 | 1.579 | 1.593 | 1.607 |
| 3.2 | 1.627 | 1.642 | 1.656 | 1.670 | 1.684 | 1.699 |
| 3.3 | 1.722 | 1.736 | 1.750 | 1.764 | 1.779 | 1.793 |
| 3.4 | 1.819 | 1.833 | 1.847 | 1.862 | 1.876 | 1.890 |
| 3.5 | 1.919 | 1.933 | 1.947 | 1.962 | 1.976 | 1.990 |
| 3.6 | 2.022 | 2.036 | 2.050 | 2.064 | 2.079 | 2.093 |
| 3.7 | 2.127 | 2.142 | 2.156 | 2.170 | 2.185 | 2.199 |
| 3.8 | 2.236 | 2.250 | 2.265 | 2.279 | 2.293 | 2.308 |
| 3.9 | 2.348 | 2.362 | 2.376 | 2.391 | 2.405 | 2.419 |
| 4.0 | 2.462 | 2.477 | 2.491 | 2.505 | 2.519 | 2.534 |
| 4.1 | 2.580 | 2.594 | 2.608 | 2.623 | 2.637 | 2.651 |
| 4.2 | 2.700 | 2.714 | 2.729 | 2.743 | 2.757 | 2.771 |
| 4.3 | 2.823 | 2.838 | 2.852 | 2.866 | 2.880 | 2.895 |
| 4.4 | 2.949 | 2.964 | 2.978 | 2.992 | 3.007 | 3.021 |
| 4.5 | 3.078 | 3.093 | 3.107 | 3.121 | 3.136 | 3.150 |
| 4.6 | 3.210 | 3.225 | 3.239 | 3.253 | 3.267 | 3.282 |
| 4.7 | 3.345 | 3.359 | 3.374 | 3.388 | 3.402 | 3.417 |
| 4.8 | 3.483 | 3.497 | 3.511 | 3.526 | 3.540 | 3.554 |
| 4.9 | 3.623 | 3.638 | 3.652 | 3.666 | 3.681 | 3.695 |
| 5.0 | 3.767 | 3.781 | 3.796 | 3.810 | 3.824 | 3.838 |

<18G>

| Flow rate  (cc/sec) | h (cm) | | | | | |
| --- | --- | --- | --- | --- | --- | --- |
|  | 10 | 11 | 12 | 13 | 14 | 15 |
| 0.1 | 0.146 | 0.160 | 0.175 | 0.189 | 0.203 | 0.217 |
| 0.2 | 0.155 | 0.169 | 0.184 | 0.198 | 0.212 | 0.226 |
| 0.3 | 0.170 | 0.184 | 0.199 | 0.213 | 0.227 | 0.242 |
| 0.4 | 0.191 | 0.206 | 0.220 | 0.234 | 0.249 | 0.263 |
| 0.5 | 0.219 | 0.233 | 0.247 | 0.262 | 0.276 | 0.290 |
| 0.6 | 0.252 | 0.266 | 0.281 | 0.295 | 0.309 | 0.323 |
| 0.7 | 0.291 | 0.306 | 0.320 | 0.334 | 0.349 | 0.363 |
| 0.8 | 0.337 | 0.351 | 0.365 | 0.380 | 0.394 | 0.408 |
| 0.9 | 0.388 | 0.403 | 0.417 | 0.431 | 0.446 | 0.460 |
| 1.0 | 0.446 | 0.460 | 0.475 | 0.489 | 0.503 | 0.517 |
| 1.1 | 0.510 | 0.524 | 0.538 | 0.553 | 0.567 | 0.581 |
| 1.2 | 0.579 | 0.594 | 0.608 | 0.622 | 0.637 | 0.651 |
| 1.3 | 0.655 | 0.669 | 0.684 | 0.698 | 0.712 | 0.727 |
| 1.4 | 0.737 | 0.751 | 0.766 | 0.780 | 0.794 | 0.809 |
| 1.5 | 0.825 | 0.839 | 0.854 | 0.868 | 0.882 | 0.896 |
| 1.6 | 0.919 | 0.933 | 0.948 | 0.962 | 0.976 | 0.990 |
| 1.7 | 1.019 | 1.033 | 1.048 | 1.062 | 1.076 | 1.090 |
| 1.8 | 1.125 | 1.139 | 1.154 | 1.168 | 1.182 | 1.197 |
| 1.9 | 1.237 | 1.252 | 1.266 | 1.280 | 1.294 | 1.309 |
| 2.0 | 1.355 | 1.370 | 1.384 | 1.398 | 1.413 | 1.427 |
| 2.1 | 1.480 | 1.494 | 1.508 | 1.523 | 1.537 | 1.551 |
| 2.2 | 1.610 | 1.624 | 1.639 | 1.653 | 1.667 | 1.682 |
| 2.3 | 1.747 | 1.761 | 1.775 | 1.789 | 1.804 | 1.818 |
| 2.4 | 1.889 | 1.903 | 1.918 | 1.932 | 1.946 | 1.960 |
| 2.5 | 2.038 | 2.052 | 2.066 | 2.080 | 2.095 | 2.109 |
| 2.6 | 2.192 | 2.206 | 2.221 | 2.235 | 2.249 | 2.264 |
| 2.7 | 2.353 | 2.367 | 2.381 | 2.396 | 2.410 | 2.424 |
| 2.8 | 2.520 | 2.534 | 2.548 | 2.562 | 2.577 | 2.591 |
| 2.9 | 2.692 | 2.707 | 2.721 | 2.735 | 2.749 | 2.764 |
| 3.0 | 2.871 | 2.885 | 2.900 | 2.914 | 2.928 | 2.943 |
| 3.1 | 3.056 | 3.070 | 3.085 | 3.099 | 3.113 | 3.128 |
| 3.2 | 3.247 | 3.261 | 3.276 | 3.290 | 3.304 | 3.319 |
| 3.3 | 3.444 | 3.458 | 3.473 | 3.487 | 3.501 | 3.516 |
| 3.4 | 3.647 | 3.661 | 3.676 | 3.690 | 3.704 | 3.719 |
| 3.5 | 3.856 | 3.871 | 3.885 | 3.899 | 3.914 | 3.928 |
| 3.6 | 4.072 | 4.086 | 4.100 | 4.114 | 4.129 | 4.143 |
| 3.7 | 4.293 | 4.307 | 4.321 | 4.336 | 4.350 | 4.364 |
| 3.8 | 4.520 | 4.535 | 4.549 | 4.563 | 4.577 | 4.592 |
| 3.9 | 4.754 | 4.768 | 4.782 | 4.797 | 4.811 | 4.825 |
| 4.0 | 4.993 | 5.007 | 5.022 | 5.036 | 5.050 | 5.065 |
| 4.1 | 5.239 | 5.253 | 5.267 | 5.282 | 5.296 | 5.310 |
| 4.2 | 5.490 | 5.505 | 5.519 | 5.533 | 5.547 | 5.562 |
| 4.3 | 5.748 | 5.762 | 5.777 | 5.791 | 5.805 | 5.819 |
| 4.4 | 6.012 | 6.026 | 6.040 | 6.055 | 6.069 | 6.083 |
| 4.5 | 6.281 | 6.296 | 6.310 | 6.324 | 6.339 | 6.353 |
| 4.6 | 6.557 | 6.572 | 6.586 | 6.600 | 6.614 | 6.629 |
| 4.7 | 6.839 | 6.854 | 6.868 | 6.882 | 6.896 | 6.911 |
| 4.8 | 7.127 | 7.142 | 7.156 | 7.170 | 7.184 | 7.199 |
| 4.9 | 7.421 | 7.436 | 7.450 | 7.464 | 7.478 | 7.493 |
| 5.0 | 7.721 | 7.736 | 7.750 | 7.764 | 7.779 | 7.793 |

<19G>

| Flow rate  (cc/sec) | h (cm) | | | | | |
| --- | --- | --- | --- | --- | --- | --- |
|  | 10 | 11 | 12 | 13 | 14 | 15 |
| 0.1 | 0.150 | 0.164 | 0.178 | 0.192 | 0.207 | 0.221 |
| 0.2 | 0.170 | 0.184 | 0.198 | 0.213 | 0.227 | 0.241 |
| 0.3 | 0.203 | 0.217 | 0.232 | 0.246 | 0.260 | 0.275 |
| 0.4 | 0.250 | 0.264 | 0.279 | 0.293 | 0.307 | 0.321 |
| 0.5 | 0.310 | 0.325 | 0.339 | 0.353 | 0.367 | 0.382 |
| 0.6 | 0.384 | 0.398 | 0.413 | 0.427 | 0.441 | 0.455 |
| 0.7 | 0.471 | 0.485 | 0.500 | 0.514 | 0.528 | 0.542 |
| 0.8 | 0.571 | 0.586 | 0.600 | 0.614 | 0.629 | 0.643 |
| 0.9 | 0.685 | 0.700 | 0.714 | 0.728 | 0.742 | 0.757 |
| 1.0 | 0.813 | 0.827 | 0.841 | 0.855 | 0.870 | 0.884 |
| 1.1 | 0.953 | 0.967 | 0.982 | 0.996 | 1.010 | 1.025 |
| 1.2 | 1.107 | 1.121 | 1.136 | 1.150 | 1.164 | 1.179 |
| 1.3 | 1.275 | 1.289 | 1.303 | 1.317 | 1.332 | 1.346 |
| 1.4 | 1.455 | 1.470 | 1.484 | 1.498 | 1.513 | 1.527 |
| 1.5 | 1.650 | 1.664 | 1.678 | 1.692 | 1.707 | 1.721 |
| 1.6 | 1.857 | 1.872 | 1.886 | 1.900 | 1.914 | 1.929 |
| 1.7 | 2.078 | 2.092 | 2.107 | 2.121 | 2.135 | 2.150 |
| 1.8 | 2.313 | 2.327 | 2.341 | 2.355 | 2.370 | 2.384 |
| 1.9 | 2.560 | 2.575 | 2.589 | 2.603 | 2.618 | 2.632 |
| 2.0 | 2.822 | 2.836 | 2.850 | 2.864 | 2.879 | 2.893 |
| 2.1 | 3.096 | 3.110 | 3.125 | 3.139 | 3.153 | 3.168 |
| 2.2 | 3.384 | 3.398 | 3.413 | 3.427 | 3.441 | 3.455 |
| 2.3 | 3.685 | 3.700 | 3.714 | 3.728 | 3.743 | 3.757 |
| 2.4 | 4.000 | 4.014 | 4.029 | 4.043 | 4.057 | 4.072 |
| 2.5 | 4.328 | 4.343 | 4.357 | 4.371 | 4.385 | 4.400 |
| 2.6 | 4.670 | 4.684 | 4.698 | 4.713 | 4.727 | 4.741 |
| 2.7 | 5.025 | 5.039 | 5.053 | 5.068 | 5.082 | 5.096 |
| 2.8 | 5.393 | 5.407 | 5.422 | 5.436 | 5.450 | 5.464 |
| 2.9 | 5.775 | 5.789 | 5.803 | 5.818 | 5.832 | 5.846 |
| 3.0 | 6.170 | 6.184 | 6.198 | 6.213 | 6.227 | 6.241 |
| 3.1 | 6.578 | 6.593 | 6.607 | 6.621 | 6.635 | 6.650 |
| 3.2 | 7.000 | 7.014 | 7.029 | 7.043 | 7.057 | 7.072 |
| 3.3 | 7.435 | 7.450 | 7.464 | 7.478 | 7.493 | 7.507 |
| 3.4 | 7.884 | 7.898 | 7.913 | 7.927 | 7.941 | 7.956 |
| 3.5 | 8.346 | 8.360 | 8.375 | 8.389 | 8.403 | 8.418 |
| 3.6 | 8.822 | 8.836 | 8.850 | 8.865 | 8.879 | 8.893 |
| 3.7 | 9.310 | 9.325 | 9.339 | 9.353 | 9.368 | 9.382 |
| 3.8 | 9.813 | 9.827 | 9.841 | 9.856 | 9.870 | 9.884 |
| 3.9 | 10.328 | 10.343 | 10.357 | 10.371 | 10.386 | 10.400 |
| 4.0 | 10.857 | 10.872 | 10.886 | 10.900 | 10.915 | 10.929 |
| 4.1 | 11.400 | 11.414 | 11.428 | 11.443 | 11.457 | 11.471 |
| 4.2 | 11.956 | 11.970 | 11.984 | 11.998 | 12.013 | 12.027 |
| 4.3 | 12.525 | 12.539 | 12.553 | 12.568 | 12.582 | 12.596 |
| 4.4 | 13.107 | 13.122 | 13.136 | 13.150 | 13.165 | 13.179 |
| 4.5 | 13.703 | 13.718 | 13.732 | 13.746 | 13.761 | 13.775 |
| 4.6 | 14.313 | 14.327 | 14.341 | 14.356 | 14.370 | 14.384 |
| 4.7 | 14.936 | 14.950 | 14.964 | 14.978 | 14.993 | 15.007 |
| 4.8 | 15.572 | 15.586 | 15.600 | 15.615 | 15.629 | 15.643 |
| 4.9 | 16.221 | 16.236 | 16.250 | 16.264 | 16.278 | 16.293 |
| 5.0 | 16.884 | 16.899 | 16.913 | 16.927 | 16.941 | 16.956 |

<20G>

| Flow rate  (cc/sec) | h (cm) | | | | | |
| --- | --- | --- | --- | --- | --- | --- |
|  | 10 | 11 | 12 | 13 | 14 | 15 |
| 0.1 | 0.155 | 0.169 | 0.183 | 0.198 | 0.212 | 0.226 |
| 0.2 | 0.191 | 0.205 | 0.220 | 0.234 | 0.248 | 0.262 |
| 0.3 | 0.251 | 0.265 | 0.280 | 0.294 | 0.308 | 0.322 |
| 0.4 | 0.335 | 0.349 | 0.364 | 0.378 | 0.392 | 0.406 |
| 0.5 | 0.443 | 0.457 | 0.472 | 0.486 | 0.500 | 0.514 |
| 0.6 | 0.575 | 0.589 | 0.604 | 0.618 | 0.632 | 0.647 |
| 0.7 | 0.731 | 0.745 | 0.760 | 0.774 | 0.788 | 0.803 |
| 0.8 | 0.911 | 0.926 | 0.940 | 0.954 | 0.968 | 0.983 |
| 0.9 | 1.115 | 1.130 | 1.144 | 1.158 | 1.173 | 1.187 |
| 1.0 | 1.344 | 1.358 | 1.372 | 1.386 | 1.401 | 1.415 |
| 1.1 | 1.596 | 1.610 | 1.624 | 1.638 | 1.653 | 1.667 |
| 1.2 | 1.872 | 1.886 | 1.900 | 1.915 | 1.929 | 1.943 |
| 1.3 | 2.172 | 2.186 | 2.200 | 2.215 | 2.229 | 2.243 |
| 1.4 | 2.496 | 2.510 | 2.525 | 2.539 | 2.553 | 2.568 |
| 1.5 | 2.844 | 2.859 | 2.873 | 2.887 | 2.901 | 2.916 |
| 1.6 | 3.216 | 3.231 | 3.245 | 3.259 | 3.274 | 3.288 |
| 1.7 | 3.613 | 3.627 | 3.641 | 3.656 | 3.670 | 3.684 |
| 1.8 | 4.033 | 4.047 | 4.061 | 4.076 | 4.090 | 4.104 |
| 1.9 | 4.477 | 4.491 | 4.506 | 4.520 | 4.534 | 4.549 |
| 2.0 | 4.945 | 4.960 | 4.974 | 4.988 | 5.002 | 5.017 |
| 2.1 | 5.438 | 5.452 | 5.466 | 5.480 | 5.495 | 5.509 |
| 2.2 | 5.954 | 5.968 | 5.982 | 5.997 | 6.011 | 6.025 |
| 2.3 | 6.494 | 6.508 | 6.523 | 6.537 | 6.551 | 6.566 |
| 2.4 | 7.058 | 7.073 | 7.087 | 7.101 | 7.116 | 7.130 |
| 2.5 | 7.647 | 7.661 | 7.675 | 7.690 | 7.704 | 7.718 |
| 2.6 | 8.259 | 8.273 | 8.288 | 8.302 | 8.316 | 8.330 |
| 2.7 | 8.895 | 8.910 | 8.924 | 8.938 | 8.952 | 8.967 |
| 2.8 | 9.556 | 9.570 | 9.584 | 9.598 | 9.613 | 9.627 |
| 2.9 | 10.240 | 10.254 | 10.269 | 10.283 | 10.297 | 10.311 |
| 3.0 | 10.948 | 10.963 | 10.977 | 10.991 | 11.005 | 11.020 |
| 3.1 | 11.681 | 11.695 | 11.709 | 11.724 | 11.738 | 11.752 |
| 3.2 | 12.437 | 12.451 | 12.466 | 12.480 | 12.494 | 12.509 |
| 3.3 | 13.217 | 13.232 | 13.246 | 13.260 | 13.275 | 13.289 |
| 3.4 | 14.022 | 14.036 | 14.050 | 14.065 | 14.079 | 14.093 |
| 3.5 | 14.850 | 14.865 | 14.879 | 14.893 | 14.907 | 14.922 |
| 3.6 | 15.703 | 15.717 | 15.731 | 15.746 | 15.760 | 15.774 |
| 3.7 | 16.579 | 16.593 | 16.608 | 16.622 | 16.636 | 16.651 |
| 3.8 | 17.480 | 17.494 | 17.508 | 17.522 | 17.537 | 17.551 |
| 3.9 | 18.404 | 18.418 | 18.433 | 18.447 | 18.461 | 18.476 |
| 4.0 | 19.353 | 19.367 | 19.381 | 19.395 | 19.410 | 19.424 |
| 4.1 | 20.325 | 20.339 | 20.354 | 20.368 | 20.382 | 20.396 |
| 4.2 | 21.322 | 21.336 | 21.350 | 21.364 | 21.379 | 21.393 |
| 4.3 | 22.342 | 22.356 | 22.371 | 22.385 | 22.399 | 22.413 |
| 4.4 | 23.387 | 23.401 | 23.415 | 23.429 | 23.444 | 23.458 |
| 4.5 | 24.455 | 24.469 | 24.484 | 24.498 | 24.512 | 24.527 |
| 4.6 | 25.548 | 25.562 | 25.576 | 25.591 | 25.605 | 25.619 |
| 4.7 | 26.664 | 26.679 | 26.693 | 26.707 | 26.721 | 26.736 |
| 4.8 | 27.805 | 27.819 | 27.833 | 27.848 | 27.862 | 27.876 |
| 4.9 | 28.969 | 28.984 | 28.998 | 29.012 | 29.027 | 29.041 |
| 5.0 | 30.158 | 30.172 | 30.187 | 30.201 | 30.215 | 30.229 |

<22G>

| Flow rate  (cc/sec) | h (cm) | | | | | |
| --- | --- | --- | --- | --- | --- | --- |
|  | 10 | 11 | 12 | 13 | 14 | 15 |
| 0.1 | 0.194 | 0.208 | 0.223 | 0.237 | 0.251 | 0.265 |
| 0.2 | 0.347 | 0.361 | 0.376 | 0.390 | 0.404 | 0.419 |
| 0.3 | 0.602 | 0.617 | 0.631 | 0.645 | 0.659 | 0.674 |
| 0.4 | 0.960 | 0.974 | 0.988 | 1.002 | 1.017 | 1.031 |
| 0.5 | 1.419 | 1.433 | 1.447 | 1.462 | 1.476 | 1.490 |
| 0.6 | 1.980 | 1.995 | 2.009 | 2.023 | 2.037 | 2.052 |
| 0.7 | 2.644 | 2.658 | 2.672 | 2.687 | 2.701 | 2.715 |
| 0.8 | 3.409 | 3.424 | 3.438 | 3.452 | 3.466 | 3.481 |
| 0.9 | 4.277 | 4.291 | 4.306 | 4.320 | 4.334 | 4.348 |
| 1.0 | 5.247 | 5.261 | 5.275 | 5.290 | 5.304 | 5.318 |
| 1.1 | 6.318 | 6.333 | 6.347 | 6.361 | 6.376 | 6.390 |
| 1.2 | 7.492 | 7.507 | 7.521 | 7.535 | 7.550 | 7.564 |
| 1.3 | 8.768 | 8.783 | 8.797 | 8.811 | 8.825 | 8.840 |
| 1.4 | 10.146 | 10.161 | 10.175 | 10.189 | 10.203 | 10.218 |
| 1.5 | 11.626 | 11.641 | 11.655 | 11.669 | 11.684 | 11.698 |
| 1.6 | 13.209 | 13.223 | 13.237 | 13.251 | 13.266 | 13.280 |
| 1.7 | 14.893 | 14.907 | 14.921 | 14.936 | 14.950 | 14.964 |
| 1.8 | 16.679 | 16.693 | 16.708 | 16.722 | 16.736 | 16.751 |
| 1.9 | 18.568 | 18.582 | 18.596 | 18.610 | 18.625 | 18.639 |
| 2.0 | 20.558 | 20.572 | 20.587 | 20.601 | 20.615 | 20.630 |
| 2.1 | 22.651 | 22.665 | 22.679 | 22.693 | 22.708 | 22.722 |
| 2.2 | 24.845 | 24.860 | 24.874 | 24.888 | 24.902 | 24.917 |
| 2.3 | 27.142 | 27.156 | 27.171 | 27.185 | 27.199 | 27.213 |
| 2.4 | 29.541 | 29.555 | 29.569 | 29.584 | 29.598 | 29.612 |
| 2.5 | 32.042 | 32.056 | 32.070 | 32.084 | 32.099 | 32.113 |
| 2.6 | 34.645 | 34.659 | 34.673 | 34.687 | 34.702 | 34.716 |
| 2.7 | 37.350 | 37.364 | 37.378 | 37.392 | 37.407 | 37.421 |
| 2.8 | 40.157 | 40.171 | 40.185 | 40.200 | 40.214 | 40.228 |
| 2.9 | 43.066 | 43.080 | 43.094 | 43.109 | 43.123 | 43.137 |
| 3.0 | 46.077 | 46.091 | 46.106 | 46.120 | 46.134 | 46.148 |
| 3.1 | 49.190 | 49.205 | 49.219 | 49.233 | 49.248 | 49.262 |
| 3.2 | 52.406 | 52.420 | 52.434 | 52.449 | 52.463 | 52.477 |
| 3.3 | 55.723 | 55.737 | 55.752 | 55.766 | 55.780 | 55.795 |
| 3.4 | 59.143 | 59.157 | 59.171 | 59.186 | 59.200 | 59.214 |
| 3.5 | 62.664 | 62.679 | 62.693 | 62.707 | 62.722 | 62.736 |
| 3.6 | 66.288 | 66.302 | 66.317 | 66.331 | 66.345 | 66.359 |
| 3.7 | 70.014 | 70.028 | 70.042 | 70.057 | 70.071 | 70.085 |
| 3.8 | 73.842 | 73.856 | 73.870 | 73.885 | 73.899 | 73.913 |
| 3.9 | 77.772 | 77.786 | 77.800 | 77.814 | 77.829 | 77.843 |
| 4.0 | 81.804 | 81.818 | 81.832 | 81.846 | 81.861 | 81.875 |
| 4.1 | 85.938 | 85.952 | 85.966 | 85.981 | 85.995 | 86.009 |
| 4.2 | 90.174 | 90.188 | 90.202 | 90.217 | 90.231 | 90.245 |
| 4.3 | 94.512 | 94.526 | 94.541 | 94.555 | 94.569 | 94.583 |
| 4.4 | 98.952 | 98.967 | 98.981 | 98.995 | 99.009 | 99.024 |
| 4.5 | 103.495 | 103.509 | 103.523 | 103.538 | 103.552 | 103.566 |
| 4.6 | 108.139 | 108.153 | 108.168 | 108.182 | 108.196 | 108.211 |
| 4.7 | 112.886 | 112.900 | 112.914 | 112.929 | 112.943 | 112.957 |
| 4.8 | 117.734 | 117.749 | 117.763 | 117.777 | 117.791 | 117.806 |
| 4.9 | 122.685 | 122.699 | 122.714 | 122.728 | 122.742 | 122.756 |
| 5.0 | 127.738 | 127.752 | 127.766 | 127.781 | 127.795 | 127.809 |

<25G>

| Flow rate  (cc/sec) | h (cm) | | | | | |
| --- | --- | --- | --- | --- | --- | --- |
|  | 10 | 11 | 12 | 13 | 14 | 15 |
| 0.1 | 0.550 | 0.565 | 0.579 | 0.593 | 0.608 | 0.622 |
| 0.2 | 1.773 | 1.787 | 1.802 | 1.816 | 1.830 | 1.845 |
| 0.3 | 3.811 | 3.825 | 3.840 | 3.854 | 3.868 | 3.882 |
| 0.4 | 6.664 | 6.678 | 6.693 | 6.707 | 6.721 | 6.735 |
| 0.5 | 10.332 | 10.346 | 10.361 | 10.375 | 10.389 | 10.404 |
| 0.6 | 14.815 | 14.830 | 14.844 | 14.858 | 14.872 | 14.887 |
| 0.7 | 20.114 | 20.128 | 20.142 | 20.157 | 20.171 | 20.185 |
| 0.8 | 26.227 | 26.242 | 26.256 | 26.270 | 26.284 | 26.299 |
| 0.9 | 33.156 | 33.170 | 33.184 | 33.199 | 33.213 | 33.227 |
| 1.0 | 40.900 | 40.914 | 40.928 | 40.943 | 40.957 | 40.971 |
| 1.1 | 49.459 | 49.473 | 49.487 | 49.501 | 49.516 | 49.530 |
| 1.2 | 58.833 | 58.847 | 58.861 | 58.875 | 58.890 | 58.904 |
| 1.3 | 69.022 | 69.036 | 69.050 | 69.065 | 69.079 | 69.093 |
| 1.4 | 80.026 | 80.040 | 80.055 | 80.069 | 80.083 | 80.098 |
| 1.5 | 91.846 | 91.860 | 91.874 | 91.888 | 91.903 | 91.917 |
| 1.6 | 104.480 | 104.494 | 104.509 | 104.523 | 104.537 | 104.552 |
| 1.7 | 117.930 | 117.944 | 117.958 | 117.973 | 117.987 | 118.001 |
| 1.8 | 132.195 | 132.209 | 132.223 | 132.238 | 132.252 | 132.266 |
| 1.9 | 147.275 | 147.289 | 147.303 | 147.318 | 147.332 | 147.346 |
| 2.0 | 163.170 | 163.184 | 163.198 | 163.213 | 163.227 | 163.241 |
| 2.1 | 179.880 | 179.894 | 179.909 | 179.923 | 179.937 | 179.952 |
| 2.2 | 197.406 | 197.420 | 197.434 | 197.448 | 197.463 | 197.477 |
| 2.3 | 215.746 | 215.760 | 215.775 | 215.789 | 215.803 | 215.818 |
| 2.4 | 234.902 | 234.916 | 234.930 | 234.945 | 234.959 | 234.973 |
| 2.5 | 254.873 | 254.887 | 254.901 | 254.915 | 254.930 | 254.944 |
| 2.6 | 275.658 | 275.673 | 275.687 | 275.701 | 275.716 | 275.730 |
| 2.7 | 297.260 | 297.274 | 297.288 | 297.302 | 297.317 | 297.331 |
| 2.8 | 319.676 | 319.690 | 319.704 | 319.719 | 319.733 | 319.747 |
| 2.9 | 342.907 | 342.921 | 342.936 | 342.950 | 342.964 | 342.979 |
| 3.0 | 366.954 | 366.968 | 366.982 | 366.996 | 367.011 | 367.025 |
| 3.1 | 391.815 | 391.829 | 391.844 | 391.858 | 391.872 | 391.887 |
| 3.2 | 417.492 | 417.506 | 417.521 | 417.535 | 417.549 | 417.563 |
| 3.3 | 443.984 | 443.998 | 444.012 | 444.027 | 444.041 | 444.055 |
| 3.4 | 471.291 | 471.305 | 471.319 | 471.334 | 471.348 | 471.362 |
| 3.5 | 499.413 | 499.427 | 499.442 | 499.456 | 499.470 | 499.484 |
| 3.6 | 528.350 | 528.365 | 528.379 | 528.393 | 528.407 | 528.422 |
| 3.7 | 558.103 | 558.117 | 558.131 | 558.146 | 558.160 | 558.174 |
| 3.8 | 588.670 | 588.685 | 588.699 | 588.713 | 588.727 | 588.742 |
| 3.9 | 620.053 | 620.067 | 620.082 | 620.096 | 620.110 | 620.124 |
| 4.0 | 652.251 | 652.265 | 652.279 | 652.294 | 652.308 | 652.322 |
| 4.1 | 685.264 | 685.278 | 685.292 | 685.307 | 685.321 | 685.335 |
| 4.2 | 719.092 | 719.106 | 719.120 | 719.135 | 719.149 | 719.163 |
| 4.3 | 753.735 | 753.749 | 753.764 | 753.778 | 753.792 | 753.807 |
| 4.4 | 789.193 | 789.208 | 789.222 | 789.236 | 789.251 | 789.265 |
| 4.5 | 825.467 | 825.481 | 825.496 | 825.510 | 825.524 | 825.538 |
| 4.6 | 862.556 | 862.570 | 862.584 | 862.598 | 862.613 | 862.627 |
| 4.7 | 900.459 | 900.474 | 900.488 | 900.502 | 900.517 | 900.531 |
| 4.8 | 939.178 | 939.193 | 939.207 | 939.221 | 939.235 | 939.250 |
| 4.9 | 978.712 | 978.727 | 978.741 | 978.755 | 978.769 | 978.784 |
| 5.0 | 1019.061 | 1019.076 | 1019.090 | 1019.104 | 1019.119 | 1019.133 |

<26G>

| Flow rate  (cc/sec) | h (cm) | | | | | |
| --- | --- | --- | --- | --- | --- | --- |
|  | 10 | 11 | 12 | 13 | 14 | 15 |
| 0.1 | 0.550 | 0.565 | 0.579 | 0.593 | 0.608 | 0.622 |
| 0.2 | 1.773 | 1.787 | 1.802 | 1.816 | 1.830 | 1.845 |
| 0.3 | 3.811 | 3.825 | 3.840 | 3.854 | 3.868 | 3.882 |
| 0.4 | 6.664 | 6.678 | 6.693 | 6.707 | 6.721 | 6.735 |
| 0.5 | 10.332 | 10.346 | 10.361 | 10.375 | 10.389 | 10.404 |
| 0.6 | 14.815 | 14.830 | 14.844 | 14.858 | 14.872 | 14.887 |
| 0.7 | 20.114 | 20.128 | 20.142 | 20.157 | 20.171 | 20.185 |
| 0.8 | 26.227 | 26.242 | 26.256 | 26.270 | 26.284 | 26.299 |
| 0.9 | 33.156 | 33.170 | 33.184 | 33.199 | 33.213 | 33.227 |
| 1.0 | 40.900 | 40.914 | 40.928 | 40.943 | 40.957 | 40.971 |
| 1.1 | 49.459 | 49.473 | 49.487 | 49.501 | 49.516 | 49.530 |
| 1.2 | 58.833 | 58.847 | 58.861 | 58.875 | 58.890 | 58.904 |
| 1.3 | 69.022 | 69.036 | 69.050 | 69.065 | 69.079 | 69.093 |
| 1.4 | 80.026 | 80.040 | 80.055 | 80.069 | 80.083 | 80.098 |
| 1.5 | 91.846 | 91.860 | 91.874 | 91.888 | 91.903 | 91.917 |
| 1.6 | 104.480 | 104.494 | 104.509 | 104.523 | 104.537 | 104.552 |
| 1.7 | 117.930 | 117.944 | 117.958 | 117.973 | 117.987 | 118.001 |
| 1.8 | 132.195 | 132.209 | 132.223 | 132.238 | 132.252 | 132.266 |
| 1.9 | 147.275 | 147.289 | 147.303 | 147.318 | 147.332 | 147.346 |
| 2.0 | 163.170 | 163.184 | 163.198 | 163.213 | 163.227 | 163.241 |
| 2.1 | 179.880 | 179.894 | 179.909 | 179.923 | 179.937 | 179.952 |
| 2.2 | 197.406 | 197.420 | 197.434 | 197.448 | 197.463 | 197.477 |
| 2.3 | 215.746 | 215.760 | 215.775 | 215.789 | 215.803 | 215.818 |
| 2.4 | 234.902 | 234.916 | 234.930 | 234.945 | 234.959 | 234.973 |
| 2.5 | 254.873 | 254.887 | 254.901 | 254.915 | 254.930 | 254.944 |
| 2.6 | 275.658 | 275.673 | 275.687 | 275.701 | 275.716 | 275.730 |
| 2.7 | 297.260 | 297.274 | 297.288 | 297.302 | 297.317 | 297.331 |
| 2.8 | 319.676 | 319.690 | 319.704 | 319.719 | 319.733 | 319.747 |
| 2.9 | 342.907 | 342.921 | 342.936 | 342.950 | 342.964 | 342.979 |
| 3.0 | 366.954 | 366.968 | 366.982 | 366.996 | 367.011 | 367.025 |
| 3.1 | 391.815 | 391.829 | 391.844 | 391.858 | 391.872 | 391.887 |
| 3.2 | 417.492 | 417.506 | 417.521 | 417.535 | 417.549 | 417.563 |
| 3.3 | 443.984 | 443.998 | 444.012 | 444.027 | 444.041 | 444.055 |
| 3.4 | 471.291 | 471.305 | 471.319 | 471.334 | 471.348 | 471.362 |
| 3.5 | 499.413 | 499.427 | 499.442 | 499.456 | 499.470 | 499.484 |
| 3.6 | 528.350 | 528.365 | 528.379 | 528.393 | 528.407 | 528.422 |
| 3.7 | 558.103 | 558.117 | 558.131 | 558.146 | 558.160 | 558.174 |
| 3.8 | 588.670 | 588.685 | 588.699 | 588.713 | 588.727 | 588.742 |
| 3.9 | 620.053 | 620.067 | 620.082 | 620.096 | 620.110 | 620.124 |
| 4.0 | 652.251 | 652.265 | 652.279 | 652.294 | 652.308 | 652.322 |
| 4.1 | 685.264 | 685.278 | 685.292 | 685.307 | 685.321 | 685.335 |
| 4.2 | 719.092 | 719.106 | 719.120 | 719.135 | 719.149 | 719.163 |
| 4.3 | 753.735 | 753.749 | 753.764 | 753.778 | 753.792 | 753.807 |
| 4.4 | 789.193 | 789.208 | 789.222 | 789.236 | 789.251 | 789.265 |
| 4.5 | 825.467 | 825.481 | 825.496 | 825.510 | 825.524 | 825.538 |
| 4.6 | 862.556 | 862.570 | 862.584 | 862.598 | 862.613 | 862.627 |
| 4.7 | 900.459 | 900.474 | 900.488 | 900.502 | 900.517 | 900.531 |
| 4.8 | 939.178 | 939.193 | 939.207 | 939.221 | 939.235 | 939.250 |
| 4.9 | 978.712 | 978.727 | 978.741 | 978.755 | 978.769 | 978.784 |
| 5.0 | 1019.061 | 1019.076 | 1019.090 | 1019.104 | 1019.119 | 1019.133 |

<27G>

| Flow rate  (cc/sec) | h (cm) | | | | | |
| --- | --- | --- | --- | --- | --- | --- |
|  | 10 | 11 | 12 | 13 | 14 | 15 |
| 0.1 | 1.173 | 1.187 | 1.202 | 1.216 | 1.230 | 1.244 |
| 0.2 | 4.263 | 4.278 | 4.292 | 4.306 | 4.320 | 4.335 |
| 0.3 | 9.414 | 9.428 | 9.442 | 9.457 | 9.471 | 9.485 |
| 0.4 | 16.625 | 16.639 | 16.653 | 16.667 | 16.682 | 16.696 |
| 0.5 | 25.895 | 25.910 | 25.924 | 25.938 | 25.953 | 25.967 |
| 0.6 | 37.227 | 37.241 | 37.255 | 37.269 | 37.284 | 37.298 |
| 0.7 | 50.618 | 50.632 | 50.646 | 50.661 | 50.675 | 50.689 |
| 0.8 | 66.069 | 66.084 | 66.098 | 66.112 | 66.127 | 66.141 |
| 0.9 | 83.581 | 83.595 | 83.610 | 83.624 | 83.638 | 83.653 |
| 1.0 | 103.153 | 103.167 | 103.182 | 103.196 | 103.210 | 103.225 |
| 1.1 | 124.785 | 124.800 | 124.814 | 124.828 | 124.842 | 124.857 |
| 1.2 | 148.478 | 148.492 | 148.506 | 148.520 | 148.535 | 148.549 |
| 1.3 | 174.230 | 174.244 | 174.259 | 174.273 | 174.287 | 174.302 |
| 1.4 | 202.043 | 202.057 | 202.071 | 202.086 | 202.100 | 202.114 |
| 1.5 | 231.916 | 231.930 | 231.944 | 231.959 | 231.973 | 231.987 |
| 1.6 | 263.849 | 263.863 | 263.878 | 263.892 | 263.906 | 263.920 |
| 1.7 | 297.842 | 297.857 | 297.871 | 297.885 | 297.899 | 297.914 |
| 1.8 | 333.896 | 333.910 | 333.924 | 333.939 | 333.953 | 333.967 |
| 1.9 | 372.010 | 372.024 | 372.038 | 372.053 | 372.067 | 372.081 |
| 2.0 | 412.184 | 412.198 | 412.212 | 412.226 | 412.241 | 412.255 |
| 2.1 | 454.418 | 454.432 | 454.446 | 454.461 | 454.475 | 454.489 |
| 2.2 | 498.712 | 498.726 | 498.741 | 498.755 | 498.769 | 498.784 |
| 2.3 | 545.067 | 545.081 | 545.095 | 545.110 | 545.124 | 545.138 |
| 2.4 | 593.482 | 593.496 | 593.510 | 593.524 | 593.539 | 593.553 |
| 2.5 | 643.956 | 643.971 | 643.985 | 643.999 | 644.014 | 644.028 |
| 2.6 | 696.492 | 696.506 | 696.520 | 696.535 | 696.549 | 696.563 |
| 2.7 | 751.087 | 751.101 | 751.116 | 751.130 | 751.144 | 751.159 |
| 2.8 | 807.743 | 807.757 | 807.771 | 807.786 | 807.800 | 807.814 |
| 2.9 | 866.458 | 866.473 | 866.487 | 866.501 | 866.516 | 866.530 |
| 3.0 | 927.234 | 927.249 | 927.263 | 927.277 | 927.292 | 927.306 |
| 3.1 | 990.071 | 990.085 | 990.099 | 990.114 | 990.128 | 990.142 |
| 3.2 | 1054.967 | 1054.981 | 1054.996 | 1055.010 | 1055.024 | 1055.039 |
| 3.3 | 1121.924 | 1121.938 | 1121.952 | 1121.967 | 1121.981 | 1121.995 |
| 3.4 | 1190.941 | 1190.955 | 1190.969 | 1190.983 | 1190.998 | 1191.012 |
| 3.5 | 1262.018 | 1262.032 | 1262.046 | 1262.060 | 1262.075 | 1262.089 |
| 3.6 | 1335.155 | 1335.169 | 1335.183 | 1335.198 | 1335.212 | 1335.226 |
| 3.7 | 1410.352 | 1410.366 | 1410.381 | 1410.395 | 1410.409 | 1410.424 |
| 3.8 | 1487.610 | 1487.624 | 1487.638 | 1487.653 | 1487.667 | 1487.681 |
| 3.9 | 1566.928 | 1566.942 | 1566.956 | 1566.971 | 1566.985 | 1566.999 |
| 4.0 | 1648.306 | 1648.320 | 1648.334 | 1648.349 | 1648.363 | 1648.377 |
| 4.1 | 1731.744 | 1731.758 | 1731.773 | 1731.787 | 1731.801 | 1731.815 |
| 4.2 | 1817.242 | 1817.257 | 1817.271 | 1817.285 | 1817.300 | 1817.314 |
| 4.3 | 1904.801 | 1904.815 | 1904.830 | 1904.844 | 1904.858 | 1904.872 |
| 4.4 | 1994.420 | 1994.434 | 1994.448 | 1994.463 | 1994.477 | 1994.491 |
| 4.5 | 2086.099 | 2086.113 | 2086.128 | 2086.142 | 2086.156 | 2086.170 |
| 4.6 | 2179.838 | 2179.852 | 2179.867 | 2179.881 | 2179.895 | 2179.910 |
| 4.7 | 2275.638 | 2275.652 | 2275.666 | 2275.681 | 2275.695 | 2275.709 |
| 4.8 | 2373.497 | 2373.512 | 2373.526 | 2373.540 | 2373.554 | 2373.569 |
| 4.9 | 2473.417 | 2473.431 | 2473.446 | 2473.460 | 2473.474 | 2473.489 |
| 5.0 | 2575.397 | 2575.412 | 2575.426 | 2575.440 | 2575.454 | 2575.469 |

<29G>

| Flow rate  (cc/sec) | h (cm) | | | | | |
| --- | --- | --- | --- | --- | --- | --- |
|  | 10 | 11 | 12 | 13 | 14 | 15 |
| 0.1 | 3.916 | 3.931 | 3.945 | 3.959 | 3.974 | 3.988 |
| 0.2 | 15.237 | 15.251 | 15.265 | 15.280 | 15.294 | 15.308 |
| 0.3 | 34.104 | 34.119 | 34.133 | 34.147 | 34.162 | 34.176 |
| 0.4 | 60.519 | 60.533 | 60.548 | 60.562 | 60.576 | 60.590 |
| 0.5 | 94.481 | 94.495 | 94.509 | 94.523 | 94.538 | 94.552 |
| 0.6 | 135.989 | 136.003 | 136.018 | 136.032 | 136.046 | 136.061 |
| 0.7 | 185.045 | 185.059 | 185.073 | 185.087 | 185.102 | 185.116 |
| 0.8 | 241.647 | 241.661 | 241.676 | 241.690 | 241.704 | 241.719 |
| 0.9 | 305.797 | 305.811 | 305.825 | 305.840 | 305.854 | 305.868 |
| 1.0 | 377.493 | 377.508 | 377.522 | 377.536 | 377.550 | 377.565 |
| 1.1 | 456.737 | 456.751 | 456.766 | 456.780 | 456.794 | 456.808 |
| 1.2 | 543.528 | 543.542 | 543.556 | 543.570 | 543.585 | 543.599 |
| 1.3 | 637.865 | 637.879 | 637.894 | 637.908 | 637.922 | 637.937 |
| 1.4 | 739.750 | 739.764 | 739.778 | 739.793 | 739.807 | 739.821 |
| 1.5 | 849.181 | 849.196 | 849.210 | 849.224 | 849.239 | 849.253 |
| 1.6 | 966.160 | 966.174 | 966.189 | 966.203 | 966.217 | 966.231 |
| 1.7 | 1090.686 | 1090.700 | 1090.714 | 1090.729 | 1090.743 | 1090.757 |
| 1.8 | 1222.758 | 1222.773 | 1222.787 | 1222.801 | 1222.815 | 1222.830 |
| 1.9 | 1362.378 | 1362.392 | 1362.407 | 1362.421 | 1362.435 | 1362.449 |
| 2.0 | 1509.545 | 1509.559 | 1509.573 | 1509.588 | 1509.602 | 1509.616 |
| 2.1 | 1664.258 | 1664.273 | 1664.287 | 1664.301 | 1664.315 | 1664.330 |
| 2.2 | 1826.519 | 1826.533 | 1826.548 | 1826.562 | 1826.576 | 1826.590 |
| 2.3 | 1996.327 | 1996.341 | 1996.355 | 1996.370 | 1996.384 | 1996.398 |
| 2.4 | 2173.681 | 2173.696 | 2173.710 | 2173.724 | 2173.739 | 2173.753 |
| 2.5 | 2358.583 | 2358.597 | 2358.612 | 2358.626 | 2358.640 | 2358.655 |
| 2.6 | 2551.032 | 2551.046 | 2551.060 | 2551.075 | 2551.089 | 2551.103 |
| 2.7 | 2751.028 | 2751.042 | 2751.056 | 2751.070 | 2751.085 | 2751.099 |
| 2.8 | 2958.570 | 2958.585 | 2958.599 | 2958.613 | 2958.627 | 2958.642 |
| 2.9 | 3173.660 | 3173.674 | 3173.689 | 3173.703 | 3173.717 | 3173.731 |
| 3.0 | 3396.297 | 3396.311 | 3396.325 | 3396.340 | 3396.354 | 3396.368 |
| 3.1 | 3626.481 | 3626.495 | 3626.509 | 3626.523 | 3626.538 | 3626.552 |
| 3.2 | 3864.211 | 3864.226 | 3864.240 | 3864.254 | 3864.269 | 3864.283 |
| 3.3 | 4109.489 | 4109.503 | 4109.518 | 4109.532 | 4109.546 | 4109.561 |
| 3.4 | 4362.314 | 4362.328 | 4362.342 | 4362.357 | 4362.371 | 4362.385 |
| 3.5 | 4622.686 | 4622.700 | 4622.714 | 4622.729 | 4622.743 | 4622.757 |
| 3.6 | 4890.605 | 4890.619 | 4890.633 | 4890.647 | 4890.662 | 4890.676 |
| 3.7 | 5166.070 | 5166.085 | 5166.099 | 5166.113 | 5166.128 | 5166.142 |
| 3.8 | 5449.083 | 5449.097 | 5449.112 | 5449.126 | 5449.140 | 5449.155 |
| 3.9 | 5739.643 | 5739.657 | 5739.672 | 5739.686 | 5739.700 | 5739.714 |
| 4.0 | 6037.750 | 6037.764 | 6037.778 | 6037.793 | 6037.807 | 6037.821 |
| 4.1 | 6343.404 | 6343.418 | 6343.432 | 6343.447 | 6343.461 | 6343.475 |
| 4.2 | 6656.605 | 6656.619 | 6656.633 | 6656.647 | 6656.662 | 6656.676 |
| 4.3 | 6977.352 | 6977.367 | 6977.381 | 6977.395 | 6977.410 | 6977.424 |
| 4.4 | 7305.647 | 7305.662 | 7305.676 | 7305.690 | 7305.704 | 7305.719 |
| 4.5 | 7641.489 | 7641.503 | 7641.518 | 7641.532 | 7641.546 | 7641.561 |
| 4.6 | 7984.878 | 7984.892 | 7984.907 | 7984.921 | 7984.935 | 7984.950 |
| 4.7 | 8335.814 | 8335.828 | 8335.843 | 8335.857 | 8335.871 | 8335.885 |
| 4.8 | 8694.297 | 8694.311 | 8694.325 | 8694.340 | 8694.354 | 8694.368 |
| 4.9 | 9060.327 | 9060.341 | 9060.355 | 9060.370 | 9060.384 | 9060.398 |
| 5.0 | 9433.904 | 9433.918 | 9433.932 | 9433.947 | 9433.961 | 9433.975 |
