# Supplementary figures and images for: Analysis of flow rate and pressure in syringe-based wound irrigation using Bernoulli's equation
Source: Sci Rep. 2022 Sep 2;12:14957. doi: 10.1038/s41598-022-19402-2 (PMC9440234; doi:10.1038/s41598-022-19402-2)

**Supplement 2.** **Flow analysis according to the height from the wound by syringe gauge**

**<14G>**


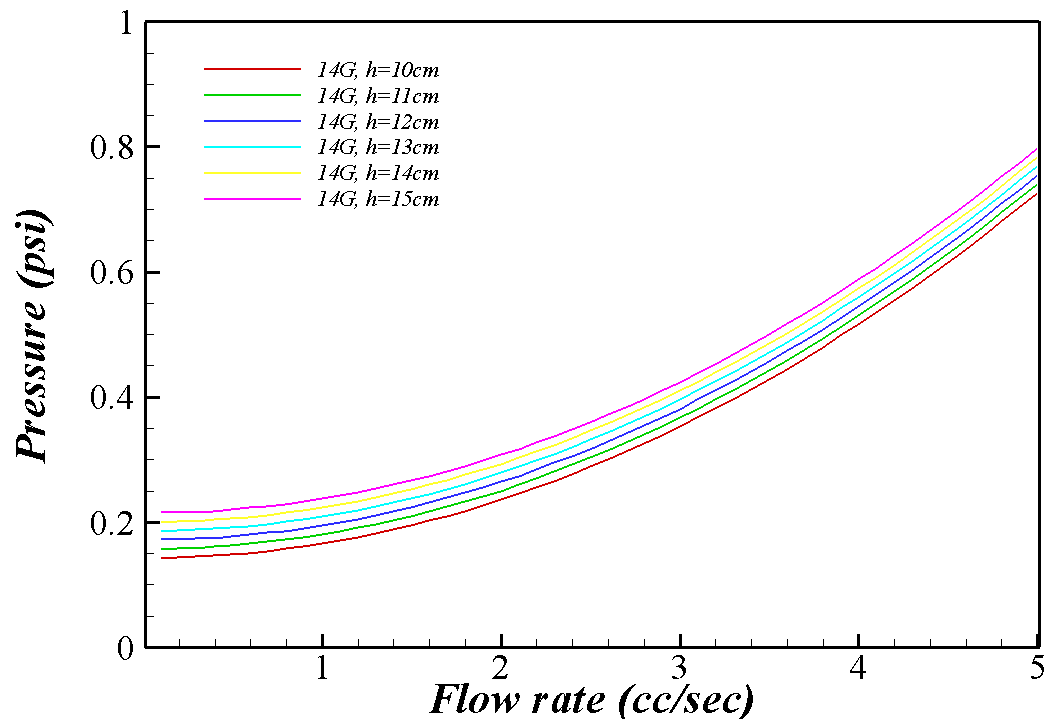


**<16G>**


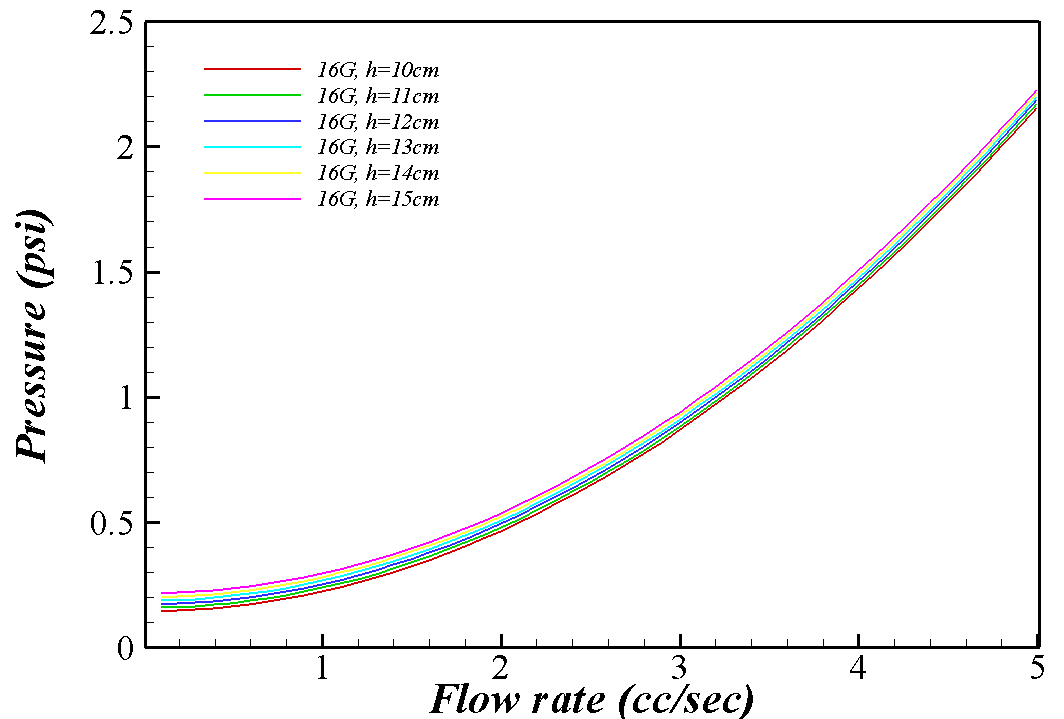


**<17G>**


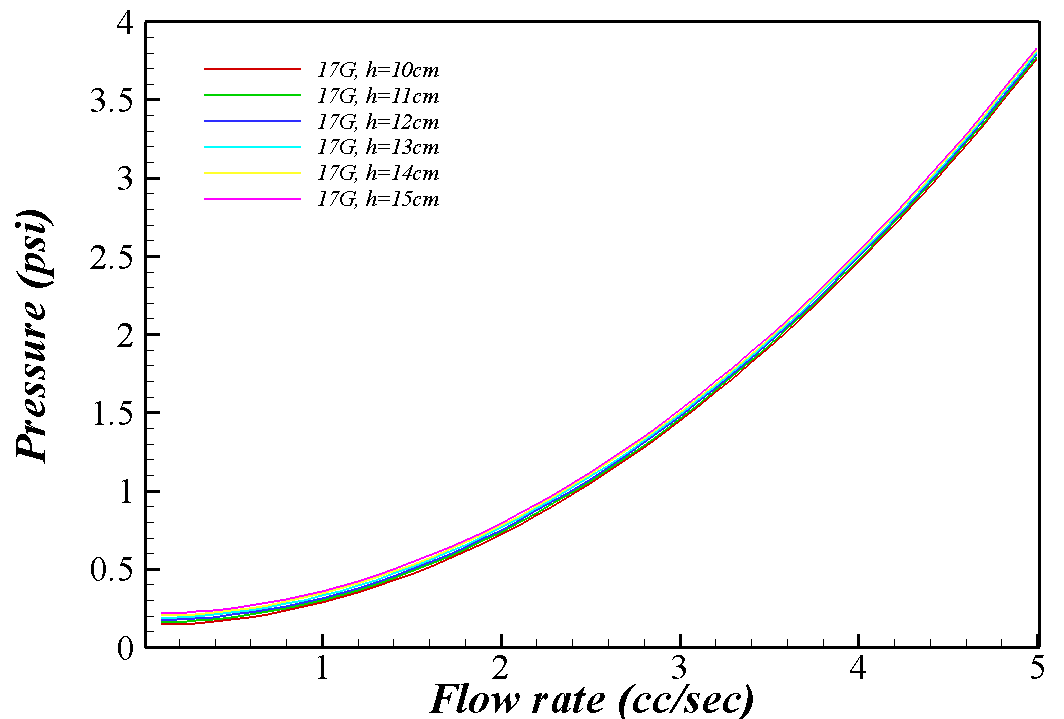


**<18G>**


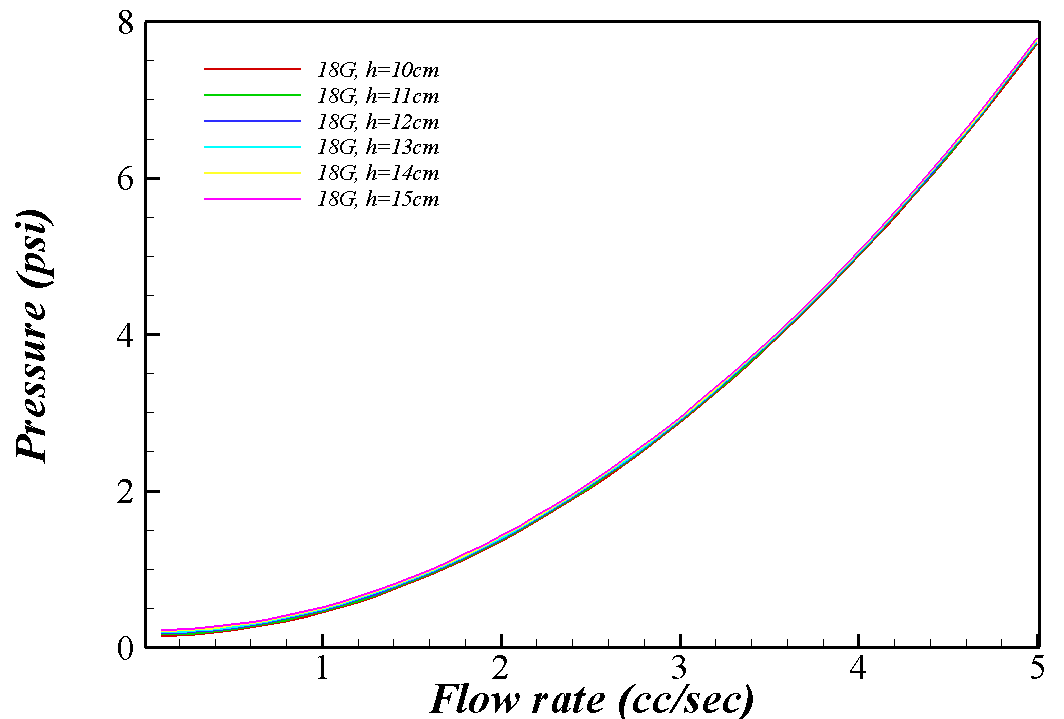


**<19G>**


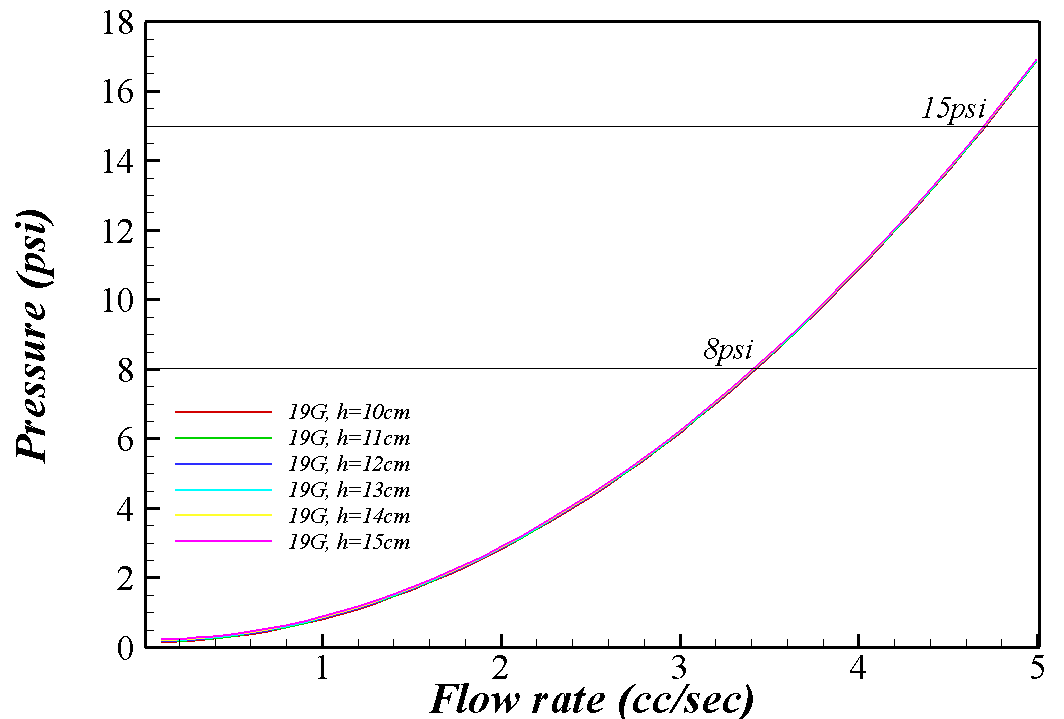


**<20G>**


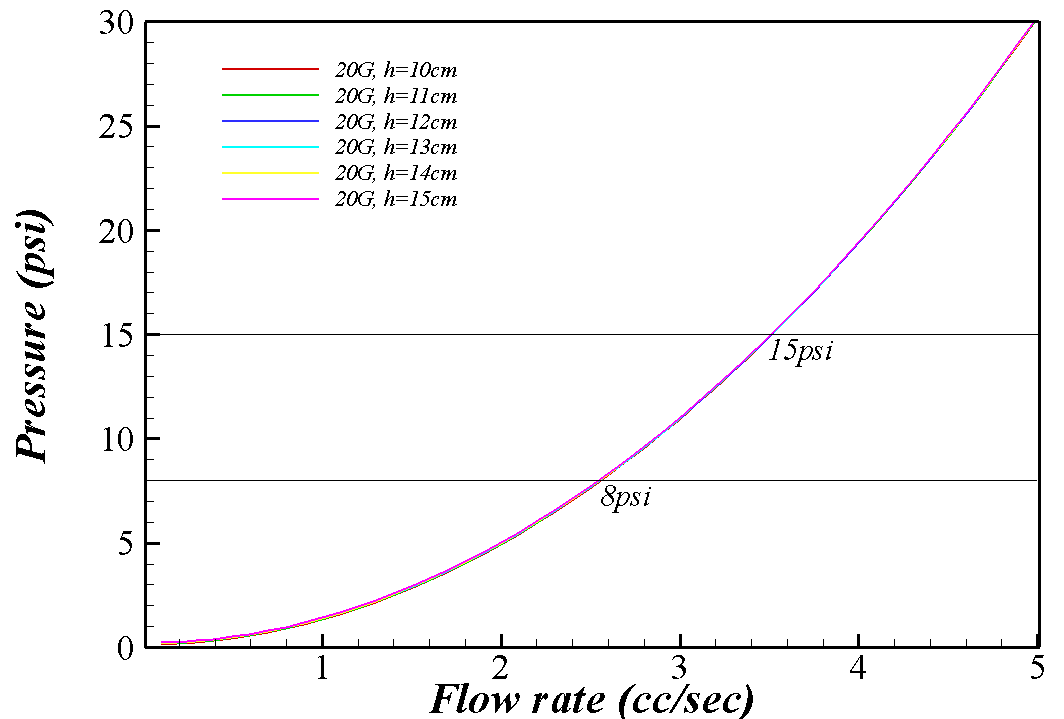


**<22G>**


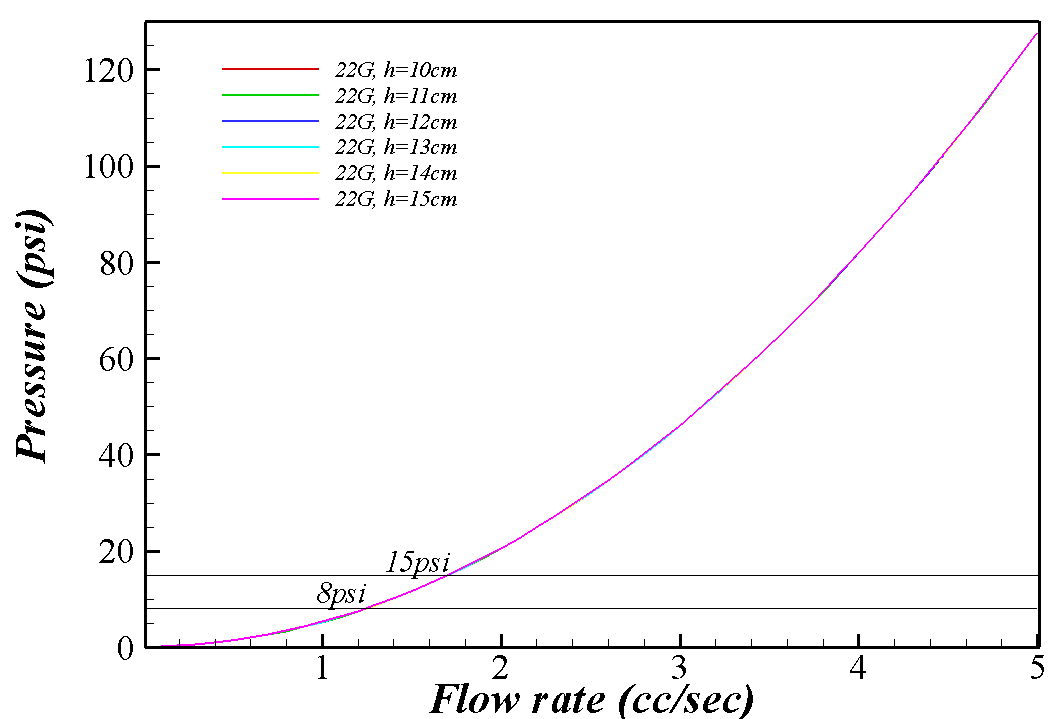


**<25G>**


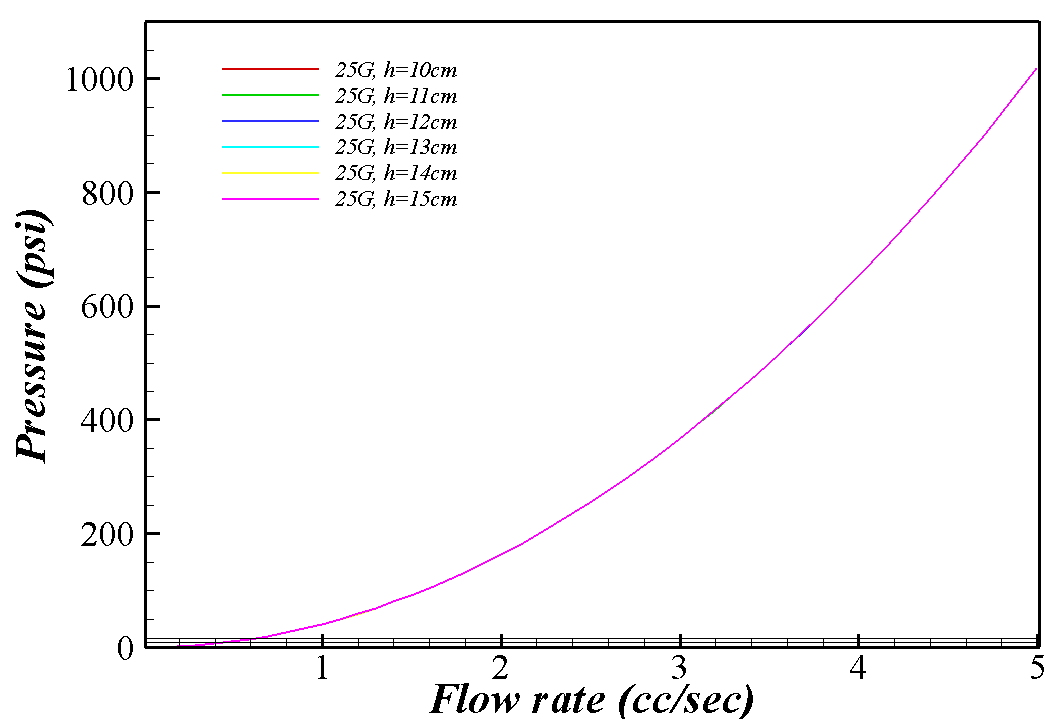


**<26G>**


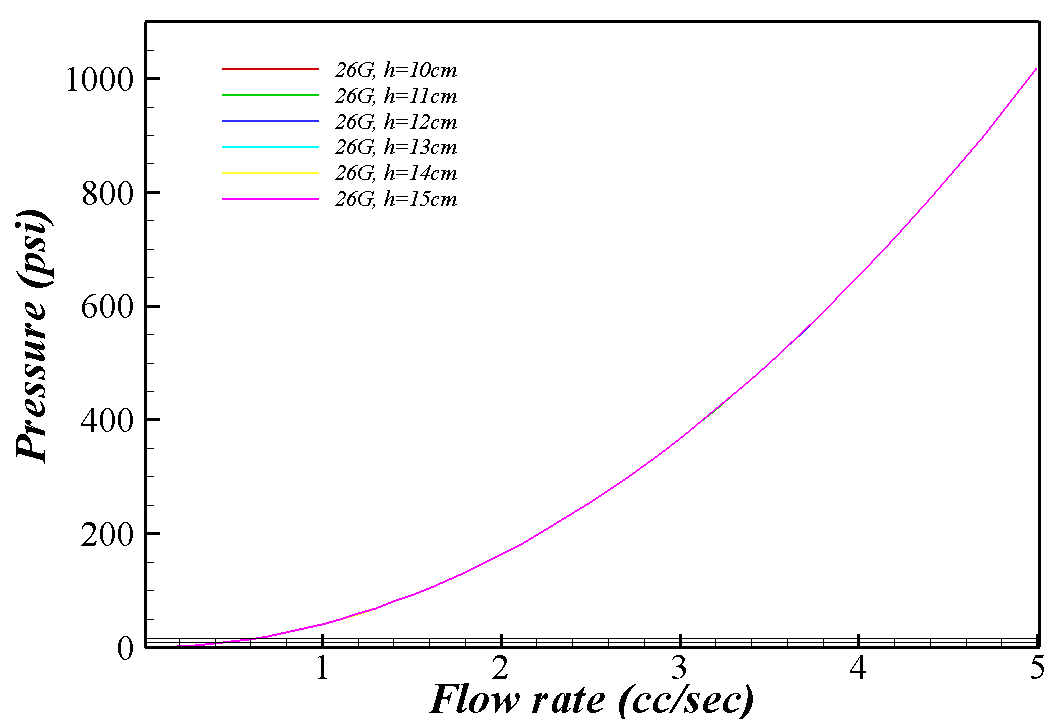


**<27G>**


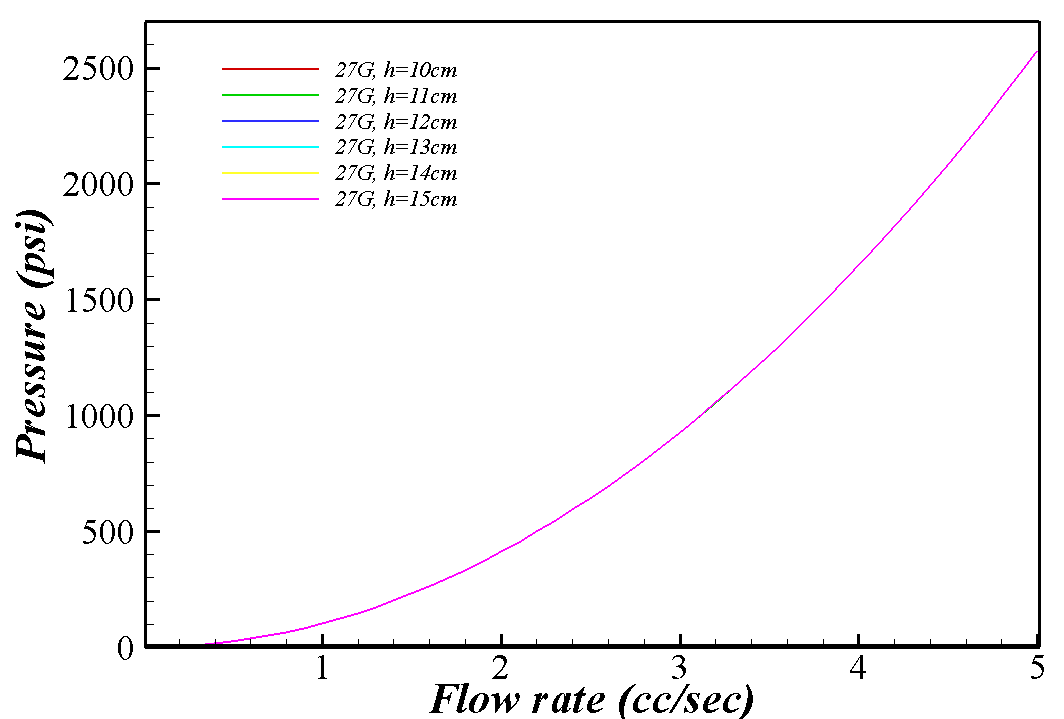


**<29G>**


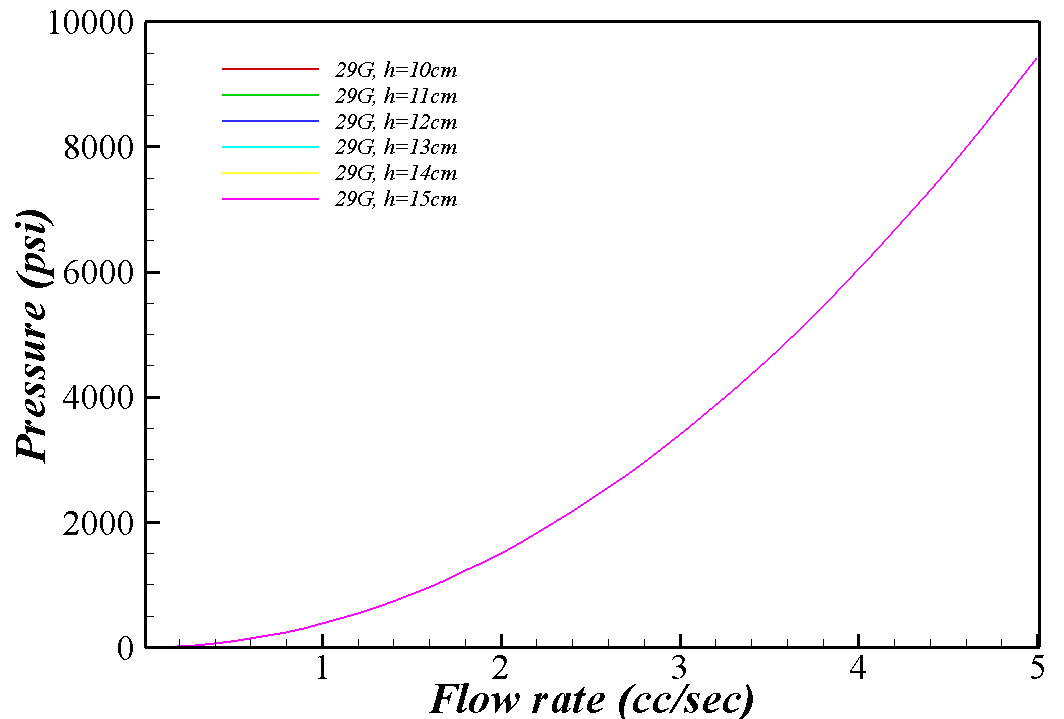

Supplement: Supplementary file 2 — Supplementary Information 2. [file 41598_2022_19402_MOESM2_ESM.docx]
